# Ultrapotent Broadly Neutralizing Human‐llama Bispecific Antibodies against HIV‐1

**DOI:** 10.1002/advs.202309268

**Published:** 2024-05-05

**Authors:** Jianliang Xu, Tongqing Zhou, Krisha McKee, Baoshan Zhang, Cuiping Liu, Alexandra F. Nazzari, Amarendra Pegu, Chen‐Hsiang Shen, Jordan E. Becker, Michael F. Bender, Payton Chan, Anita Changela, Ridhi Chaudhary, Xuejun Chen, Tal Einav, Young Do Kwon, Bob C. Lin, Mark K. Louder, Jonah S. Merriam, Nicholas C. Morano, Sijy O'Dell, Adam S. Olia, Reda Rawi, Ryan S. Roark, Tyler Stephens, I‐Ting Teng, Emily Tourtellott‐Fogt, Shuishu Wang, Eun Sung Yang, Lawrence Shapiro, Yaroslav Tsybovsky, Nicole A. Doria‐Rose, Rafael Casellas, Peter D. Kwong

**Affiliations:** ^1^ Vaccine Research Center National Institute of Allergy and Infectious Diseases National Institutes of Health Bethesda MD 20892 USA; ^2^ Laboratory of Lymphocyte Nuclear Biology National Institute of Arthritis and Musculoskeletal and Skin Diseases NIH Bethesda MD 20892 USA; ^3^ Department of Biology Georgia State University Atlanta GA 30303 USA; ^4^ Zuckerman Mind Brain Behavior Institute Columbia University New York NY 10027 USA; ^5^ Department of Biochemistry and Molecular Biophysics Columbia University Vagelos College of Physicians and Surgeons New York NY 10032 USA; ^6^ Center for Vaccine Innovation La Jolla Institute for Immunology La Jolla CA 92037 USA; ^7^ Electron Microscopy Laboratory Cancer Research Technology Program Leidos Biomedical Research Frederick National Laboratory for Cancer Research Frederick MD 21702 USA; ^8^ Aaron Diamond AIDS Research Center Columbia University Vagelos College of Physicians and Surgeons New York NY 10032 USA; ^9^ Hematopoietic Biology and Malignancy MD Anderson Cancer Center Houston TX 77054 USA

**Keywords:** bispecific antibodies, bNAb, broadly neutralizing antibody, HIV‐1, llama, neutralizing nanobodies, vaccination, vaccine

## Abstract

Broadly neutralizing antibodies are proposed as therapeutic and prophylactic agents against HIV‐1, but their potency and breadth are less than optimal. This study describes the immunization of a llama with the prefusion‐stabilized HIV‐1 envelope (Env) trimer, BG505 DS‐SOSIP, and the identification and improvement of potent neutralizing nanobodies recognizing the CD4‐binding site (CD4bs) of vulnerability. Two of the vaccine‐elicited CD4bs‐targeting nanobodies, G36 and R27, when engineered into a triple tandem format with llama IgG2a‐hinge region and human IgG1‐constant region (G36×3‐IgG2a and R27×3‐IgG2a), neutralized 96% of a multiclade 208‐strain panel at geometric mean IC_80_s of 0.314 and 0.033 µg mL^−1^, respectively. Cryo‐EM structures of these nanobodies in complex with Env trimer revealed the two nanobodies to neutralize HIV‐1 by mimicking the recognition of the CD4 receptor. To enhance their neutralizing potency and breadth, nanobodies are linked to the light chain of the V2‐apex‐targeting broadly neutralizing antibody, CAP256V2LS. The resultant human‐llama bispecific antibody CAP256L‐R27×3LS exhibited ultrapotent neutralization and breadth exceeding other published HIV‐1 broadly neutralizing antibodies, with pharmacokinetics determined in FcRn‐Fc mice similar to the parent CAP256V2LS. Vaccine‐elicited llama nanobodies, when combined with V2‐apex broadly neutralizing antibodies, may therefore be able to fulfill anti‐HIV‐1 therapeutic and prophylactic clinical goals.

## Graphical abstract

This study describes the immunization of a llama with the prefusion‐stabilized HIV‐1 envelope trimer, BG505 DS‐SOSIP, and the identification and improvement of potent neutralizing nanobodies recognizing the CD4‐binding site of vulnerability. Two human‐llama bispecific antibodies, CAP256L‐G36×3LS and CAP256L‐R27×3LS, are engineered, exhibit ultrapotent HIV‐1 neutralization and breadth, and may therefore fulfill anti‐HIV‐1 clinical goals.

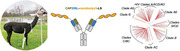

## 1 Introduction

Nanobodies are antigen‐binding entities typically derived from the heavy chain‐only antibodies of camelid animals,^[^ ^1^ ^]^ and they retain full antigen specificity with a single antibody domain.^[^ ^2^ ^]^ They can penetrate tissues and recognize epitopes that are often inaccessible to conventional antibodies.^[^ ^2^ ^]^ Such “hidden” epitopes also have the potential to be conserved across viral strains, making nanobodies ideal antiviral molecules with great cross‐reactivity to multiple viral pathogens. HIV‐1 broadly neutralizing antibodies (bNAbs) target vulnerable epitopes on the envelope (Env) trimer, such as the CD4‐binding site (CD4bs), V2‐apex, V3‐glycan, and fusion peptide (FP).^[^ ^3^ ^]^ However, it usually takes years for bNAbs to develop as they need to overcome extensive barriers to acquire the ability to target epitopes beneath the Env glycan shield.^[^ ^4^ ^]^ In contrast, nanobodies may be able to overcome such barriers more easily by virtue of their extended CDR3s and single domain character, which enables them to access Env crevices.

Indeed, a broad and potent nanobody J3 has been identified from gp140 immunization in llama and shown to mimic CD4 binding.^[^ ^5^ ^]^ Recently, in a proof of concept study, we created a bispecific antibody that fuses J3 to the light chain of the potentV2‐apex antibody CAP256V2LS and observed synergistic neutralization between J3 and CAP256V2LS, with substantially improved breadth and potency.^[^ ^6^ ^]^ Several other bispecific and trispecific antibodies have also been reported, such as VRC01‐PGDM1400‐10E8v4,^[^ ^7^ ^]^ N6‐PGDM1400‐10E8v4,^[^ ^7^ ^]^ and Tri‐Nab,^[^ ^8^ ^]^ which achieve near pan neutralization of a 208‐strain panel with geometric mean IC_80_s of ≈0.15 µg mL^−1^.

We have previously designed an HIV‐1 Env trimer BG505 DS‐SOSIP, a highly desirable antigen that is conformationally fixed in a prefusion‐closed state, in which neutralizing epitopes are almost exclusively exposed and non‐neutralizing or poorly neutralizing epitopes are hidden, even in the presence of CD4.^[^ ^9^ ^]^ In a recent clinical trial evaluating its safety and immunogenicity, we found that three injections of BG505 DS‐SOSIP elicited binding antibodies against trimer immunogen in all groups, however, most of these antibodies targeted the glycan‐free trimer base.^[^ ^10^ ^]^ From one donor, by sorting B cells that can bind to glycan‐base covered BG505 trimer, we identified autologous neutralizing antibodies against the fusion‐peptide site of vulnerability.^[^ ^11^ ^]^ In this study, we explore the ability of BG505 DS‐SOSIP to elicit a neutralizing response in llama by repetitive immunizations and isolate broadly neutralizing nanobodies using Env trimer and subdomains of trimer. We further explore generalizable strategies for nanobody improvement and combine the most potent nanobodies with CAP256V2LS to create ultrapotent human‐llama bispecific antibodies against HIV‐1.

## 2 Results

### 2.1 BG505 DS‐SOSIP Immunized Llama Develops Broadly Neutralizing Serum Responses

**Figure 1:**
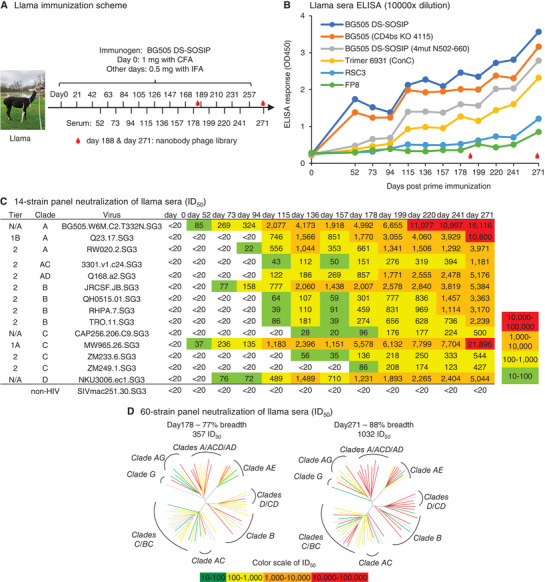
BG505 DS‐SOSIP immunized llama develops broadly neutralizing serum responses. A) Immunization schema in a llama. The llama was immunized with BG505 DS‐SOSIP subcutaneously once every 21 days (except the last boost) as indicated above the arrow line, and serum was collected 10 days post each immunization starting from day 52 as indicated beneath the arrow line. B) Antibody binding response to HIV Env probes in the immunized llama as determined by ELISA. C) Llama sera neutralization response on a 14‐strain panel. Color shading represents potency as indicated on the right of the table. D) Dendrograms of the neutralization activity of day 188 and day 271 sera on a 60‐strain panel. Dendrograms display the diversity of tested viral strains, with branches colored according to neutralization potency (non‐neutralized branches shown in gray).

To explore the anti‐HIV potential of nanobodies, we immunized a llama with prefusion‐stabilized HIV‐1 Env trimer BG505 DS‐SOSIP and sought to isolate broadly HIV‐1 neutralizing nanobodies. We immunized the llama 13 times (**Figure** **1**A) and monitored the serum antibody response against multiple HIV Env probes over the course of 271 days (Figure 1B). The immunization quickly induced strong autologous sera titer, as well as slightly weaker titer against BG505 (CD4bs KO 4115), the CD4bs epitope knocked out version of BG505 DS‐SOSIP, indicating the existence of CD4bs directed response on BG505 DS‐SOSIP. The antibody responses were weaker against glycan base‐covered Env trimer BG505 DS‐SOSIP (4mut N502‐660) at early time points, suggesting that the early immune response was primarily directed against the exposed base region of the soluble HIV‐1 Env trimer. This result aligns well with the almost exclusive glycan‐free trimer base‐targeting antibody responses observed in the 3‐dose clinical trial study. However, starting from day ≈115, a stronger response developed against the glycan‐base covered Env trimer and Env of ConC strain, suggestive of non‐base epitope targeting and cross‐clade recognition. Interestingly, epitope‐specific antibody responses against the CD4bs‐specific probe RSC3^[^ ^3i^ ^]^ and the fusion peptide‐specific probe FP8^[^ ^12^ ^]^ were not detected until later time points in the immunization.

Consistent with ELISA results at days 52, 73, and 94 showing an early response primarily focused on the Env trimer base, the serum neutralizing potency and breadth on a 14‐strain panel were weak at these three time points, and the serum neutralization response improved substantially starting on day 115 (Figure 1C). The neutralization breadth reached 100% by day 178, and the potency against the tested 14 strains continued to increase until the termination of the experiment on day 271. We further tested the neutralization activity of the sera from days 178 and 271 on an unbiased 50‐strain panel.^[^ ^13^ ^]^ The data on the non‐overlapping 60‐strains from the two panels showed that the neutralization breadth increased from 77% on day 178 to 88% by day 271, and the neutralization titer (geometric mean ID_50_) increased from 357 on day 178 to 1032 on day 271 (Figure 1D; Figure S1A, Supporting Information). Neutralization fingerprinting analysis^[^ ^14^ ^]^ predicted that the majority of the neutralization was VRC01‐like (66%–70%), indicating that CD4bs‐directed antibody response was dominant (Figure S1B, Supporting Information). These results demonstrated that BG505 DS‐SOSIP can elicit a broad and potent anti‐HIV‐1 antibody response in llama within a short period of time.

### 2.2 Isolation of HIV‐1 Neutralizing Nanobodies

**Figure 2:**
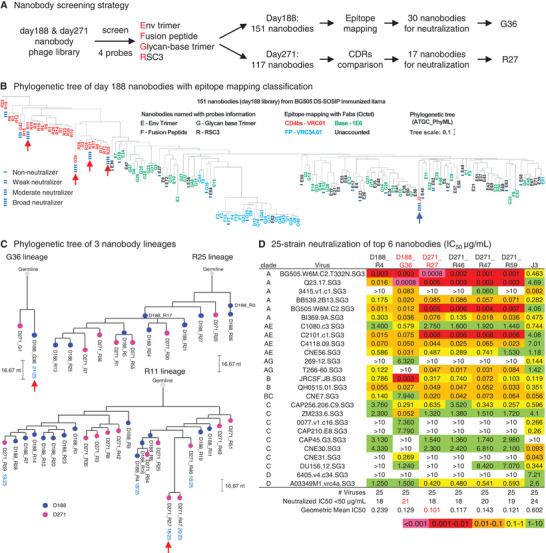
Identification of neutralizing nanobodies from BG505 DS‐SOSIP immunized llama. A) Nanobody phage library construction and screening. The four probes used for phage screening are: Env Trimer (BG505 DS‐SOSIP), Fusion peptide, Glycan base trimer (BG505 DS‐SOSIP.4mut_N502‐660), and RSC3. B) Summary of epitopes of 151 nanobodies from day 188 library. 30 nanobodies selected for small panel neutralization test are grouped into 4 categories: Non‐neutralizer (one line); Weak neutralizer (two lines); Moderate neutralizer (three lines); Broad neutralizer (four lines plus one red arrow). Control nanobody J3 is marked with four lines and one blue arrow. C) Phylogenetic tree of three selected nanobody lineages. 42 nanobodies from the three lineages were tested on a 10‐strain panel first, then the top 6 candidates were further tested on an additional 15‐strain panel. Neutralization breadth of the top 6 nanobodies on a 25‐strain panel is shown beneath the nanobody names. Scale bars indicate the distance of 16.67 nucleotides (nt) in each tree. D) 25‐strain neutralization of the top 6 nanobodies from Figure S3C (Supporting Information). The broadest (G36) and most potent (R27) nanobodies were selected for further analysis.

To isolate monoclonal neutralizing nanobodies, we constructed a nanobody phage library from the PBMCs of the llama on day 188, which is 10 days after serum neutralizing breadth first reached 100% on a 14‐strain panel (**Figure** **2**A). Four protein/peptide probes were used for nanobody screening: Env Trimer (BG505 DS‐SOSIP), Fusion peptide, Glycan‐base trimer (BG505 DS‐SOSIP.4mut_N502‐660), and RSC3. The nanobodies identified using these probes were named after the first letter of each corresponding probe name (underlined in the probe name) followed by a number. In total, 151 nanobodies with unique sequences were identified and expressed for further analysis. The binding epitopes on HIV‐1 Env for these nanobodies were mapped by a competition Biolayer interferometry (BLI) assay using Fabs of five antibodies with known epitopes: VRC01^[^ ^3i^ ^]^ for the CD4bs, VRC34.01^[^ ^3e^ ^]^ for the FP epitope, 1E6 (also called RM19R)^[^ ^15^ ^]^ for the Env‐base, PGT145^[^ ^16^ ^]^ for the V2‐apex, and 10–1074^[^ ^17^ ^]^ for the V3 glycan (Figure S2A,B, Supporting Information). Of the 69 nanobodies identified by the Env trimer probe, 4 competed with VRC34.01, 19 competed with 1E6, and 46 did not compete with any of the five antibodies. Of the 9 nanobodies identified with the FP probe, 7 competed with VRC34.01, and 2 competed with 1E6. Of the 45 nanobodies identified with the Glycan‐base trimer, 2 competed with VRC01, 17 competed with VRC34.01, and 26 competed with 1E6. Interestingly, all 28 nanobodies identified with the RSC3 probe competed with VRC01, indicating that these nanobodies are CD4bs‐targeting. More importantly, phylogenetic analysis of the 151 Env‐binding nanobodies showed that VRC01‐competing nanobodies and VRC34.01‐competing nanobodies formed two distinct clusters (Figure 2B). Based on the phylogenetic tree, we selected 30 representative nanobodies, 10 VRC34.01‐ or 1E6‐competing, 14 non‐competing, and 6 VRC01‐competing, and tested their neutralization activity on the same 14‐strain panel used for the initial serum test. None of the 10 VRC34.01‐ or 1E6‐competing nanobodies neutralized any strain (non‐neutralizers). Only 1 of the 14 non‐competing nanobodies neutralized 2 strains (weak neutralizers) (Figure 2B; Figure S2C, Supporting Information). However, the 6 VRC01‐competing nanobodies neutralized these 14 strains, with various potency and breadth (Figure S2D, Supporting Information). Specifically, we identified 4 broad neutralizers. G36 neutralized all 14 strains with a geometric mean IC_50_ of 0.088 µg mL^−1^; R11, R21, and R25 neutralized over 11 strains out of 14; and two moderate neutralizers, G42 and R18, neutralized 8 strains out of 14.

We next constructed a second nanobody phage library from the day 271 PBMCs, carried out the same screening, and identified 117 new nanobodies with unique amino acid sequences. By comparing their complementarity‐determining region (CDR) sequences, day 271 nanobodies that were similar to day 188 non‐neutralizers or weak neutralizers were removed from further analysis. From the remaining nanobodies, 17 representative ones were selected for neutralization assessment on a 5‐strain panel that included representative clades in the above‐mentioned 14‐strain panel (Figure S3A,B, Supporting Information). Among these 17 nanobodies, R27, E46 and G1 neutralized 5, 2, and 4 strains, respectively.

### 2.3 Three Lineages of HIV‐1 Neutralizing Nanobodies

To obtain a more comprehensive analysis of potentially potent and broadly neutralizing nanobodies, we plotted phylogenetic trees for 42 nanobodies identified from the day 188 and day 271 libraries. These nanobodies belong to the same lineages of the 4 broad neutralizers identified from the day 188 library, namely G36, R11, R21, and R25 (Figure 2C). The D188_G36 and D271_G1 nanobodies formed a small lineage; the R11 lineage included 13 nanobodies isolated from day 188 library and 12 nanobodies from day 271 library, including D271_R27; the R21 and R25 belonged to the same lineage along with 7 other nanobodies from day 188 and 6 nanobodies from day 271. These 42 nanobodies showed diverse neutralizing breadth and potency on a 10‐strain panel, and 6 of them showed 90% breadth (Figure S3C, Supporting Information); these 6 nanobodies were tested on an additional 15‐strain panel. The broadest neutralizing nanobody D188_G36 (21/25, hereafter referred to as G36) and the most potent neutralizing nanobody D271_R27 (geometric mean IC_50_: 0.101 µg mL^−1^, hereafter referred to as R27) were selected for further characterization (Figure 2D). Of note, G36 and R27 are 5–6 fold more potent in neutralizing the multiclade 25‐strain panel than the previously reported nanobody J3.

### 2.4 Improved Neutralization Potency and Breadth by Fc Conjugation and Multimerization

**Figure 3:**
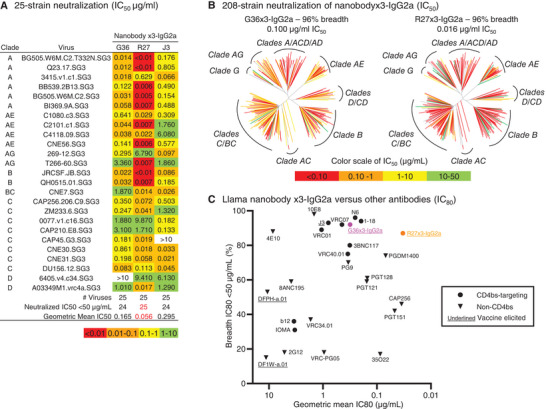
Immunization‐elicited nanobodies, G36 and R27, in nanobody x3‐IgG2a format, show broad and potent HIV‐1 neutralization. A) 25‐strain neutralization of nanobody x3‐IgG2a. B) 208‐strain panel neutralization of G36×3‐IgG2a and R27×3‐IgG2a. Dendrograms display the diversity of tested viral strains, with branches colored according to neutralization potency (non‐neutralized branches shown in gray). IC_50_ shown is geometric mean. C) Comparison of neutralization breadth and potency for G36×3‐IgG2a and R27×3‐IgG2a with other human antibodies and vaccine‐elicited NHP antibodies on 208‐strain panel.

As multimerization and Fc conjugation can increase the apparent affinity of nanobodies and their neutralization potency,^[^ ^18^ ^]^ we first carried out such engineering for G36 and R27. A 5‐strain panel test revealed that the potency of both nanobodies was improved by Fc conjugation (bivalent) based on the molarity of the whole molecules and was further improved by combining tandem multimerization and Fc conjugation (nanobody x3‐IgG2a) (Figure S4A–C, Supporting Information). Of note, nanobody x3‐IgG2a configuration enhanced the potency of R27 more than G36, indicating that the two nanobodies may neutralize HIV‐1 via different mechanisms. Similar potency improvement patterns were seen for several other less potent and less broadly neutralizing nanobodies. Most interestingly, three VRC34.01‐competing non‐neutralizing nanobodies, F7, G25, and G39, became neutralizers of 1 or 3 strains after such modification, suggesting that Fc conjugation and nanobody multimerization can be a generalizable strategy for nanobody improvement (Figure S4D, Supporting Information). The improved breadth and potency of G36×3‐IgG2a and R27×3‐IgG2a over their monomeric (single domain) forms were further confirmed in the multiclade 25‐strain panel (**Figure** **3**A), with G36×3‐IgG2a reaching 96% breadth, R27×3‐IgG2a reaching 100% breadth, and both reaching higher potency than J3×3‐IgG2a. The standard 208‐strain panel assay was then performed on the two antibodies in x3‐IgG2a format. The results showed that both nanobodies neutralized 96% of the panel at geometric mean IC_50_s of 0.1 and 0.016 µg mL^−1^ for G36×3‐IgG2a and R27×3‐IgG2a, respectively (Figure 3B, with explicit IC_50_ and IC_80_ values provided in Dataset S1, Supporting Information). The breadth of 92% and 87% (with IC_80_<50 µg mL^−1^) and geometric mean IC_80_s of 0.314 and 0.033 µg mL^−1^ for G36×3‐IgG2a and R27×3‐IgG2a, respectively (Figure 3C; Figure S4E and Dataset S1, Supporting Information), to our knowledge, rank among the best reported HIV‐1 broadly neutralizing antibodies and nanobodies.

To gain a better understanding of how the HIV‐1 Env trimer interacts with nanobodies and nanobody x3‐IgG2a, we performed a BLI experiment to determine the binding kinetics of BG505 DS‐SOSIP to immobilized nanobody molecules, G36, R27, G36×3‐IgG2a, and R27×3‐IgG2a (Figure S4F, Supporting Information). By immobilizing nanobody molecules, we created multiple BG505 DS‐SOSIP binding moieties on biosensors for G36 and R27, enabling comparison to G36×3‐IgG2a and R27×3‐IgG2a. The association and disassociation curves of all four samples could only be modeled by 1:2 (bivalent) fitting, an indication that a single BG505 DS‐SOSIP molecule could indeed bind to multiple nanobody units on biosensors. The association rate of BG505 DS‐SOSIP to all four molecules was similar, indicating that nanobody units of nanobody x3 versions function equally as nanobody monomers. The BG505 DS‐SOSIP disassociation rate was slightly reduced in nanobody x3 format, suggesting the effect of avidity was empowered by the multiple binding units on the molecules.

### 2.5 Structural Basis for the Broad HIV‐1 Neutralization of R27 and G36

**Figure 4:**
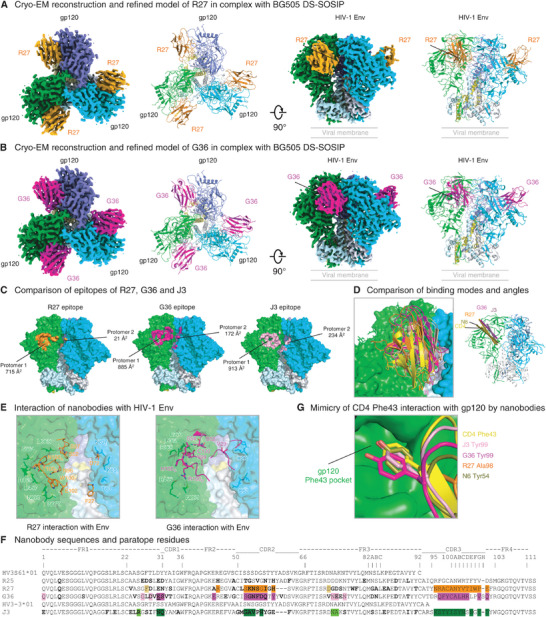
Cryo‐EM structures of nanobodies R27 and G36 in complex with HIV‐1 Env trimer reveal modes of recognition similar to J3. A) Cryo‐EM structure of nanobody R27 in complex with HIV‐1 BG505 DS‐SOSIP Env. Overall cryo‐EM density map and refined model are shown in two views with gp120 protomers colored green, cyan, and slate, respectively. The density and model of nanobody R27 is colored orange. The contour level of Cryo‐EM map is 9.5 σ. B) Cryo‐EM structure of VHH G36 in complex with HIV‐1 BG505 DS‐SOSIP Env. Overall cryo‐EM density map and refined model are shown in two views with gp120 protomers colored green, cyan, and slate, respectively. The density and model of G36 is colored magenta. The contour level of Cryo‐EM map is 9.6 σ. C) Epitopes of R27 and G36 on BG505 DS‐SOSIP. Epitopes of R27, G36, and J3 are shown in orange, magenta, and pink surfaces, respectively. R27 has a much smaller contact area on the neighboring protomer. D) Comparison of binding modes and angles. (Left) Structures of nanobodies R27, G36, and J3 are aligned with CD4 by the gp120 domain shown in green. R27, G36, and J3 are roughly in a similar position with N termini (labeled with “N”) in close proximity. (Right) The axes of R27, G36, and J3 are shown in orange, magenta, and pink rods. Axes of CD4 domain 1 and Fv domain of VRC01‐class antibody N6 are shown in yellow and olive rods for comparison. E) Detailed interactions between nanobodies and BG505 DS‐SOSIP. Residues that form hydrogen bonds and salt bridges are highlighted with sticks representation with bonds between atoms shown in gray dotted lines. Nanobodies and protomers of HIV Env are colored the same as in panels A and B. F) Alignment of nanobody sequences. Paratope residues are colored in orange and magenta, respectively. Residues interacting with neighboring protomer are colored in lighter shades. Residues interacting with both protomers are underlined. G) Nanobody mimicry of CD4 Phe43 interaction with gp120. G36, like J3, inserts Tyr99 into the “Phe43 pocket” on gp120, whereas R27 has an Ala at this position.

To elucidate the mechanism by which the potent and broad llama nanobodies recognized the Env trimer, we determined the structures of R27 and G36 in complex with BG505 DS‐SOSIP by cryo‐EM. The cryo‐EM reconstruction map of R27 in complex with BG505 DS‐SOSIP was obtained at 3.6 Å nominal resolution from 315969 particles (**Figure** **4**A; Figure S5 and Table S1, Supporting Information). The refined structural model revealed the binding of three copies of R27, each to a protomer of the Env trimer, at the CD4bs. Cryo‐EM reconstruction of G36 in complex with BG505 DS‐SOSIP was achieved at 3.3 Å from 414002 particles, and the refined structure revealed a binding mode of G36 similar to that of R27 (Figure 4B; Figure S6 and Table S1, Supporting Information). The epitope of R27 covered 715 Å^2^ on one protomer with a minor interaction of 21 Å^2^ with a neighboring protomer (Figure 4C; Figure S7, Supporting Information). The G36 epitope was slightly larger, covering 885 Å^2^ on one protomer and 172 Å^2^ on the neighboring protomer (Figure 4C; Figure S8, Supporting Information). Both epitopes were at a similar location to that of J3.^[^ ^5^, ^19^ ^]^ Indeed, R27, G36, and J3, as well as the domain 1 of CD4, are all bound at a similar location in the canyon between two gp120 subunits (Figure 4D). The approach angles of R27 and G36 were different (Figure 4D). This and their different sequences resulted in differences in paratope‐epitope interactions between the two nanobody‐Env trimer complexes. Both R27 and G36 made extensive interactions with the CD4‐binding loop, loops D and V5 on the major binding gp120 protomer through residues in their CDR2 and CDR3 (Figure 4E,F; Figures S7 and S8, Supporting Information), typical for CD4bs‐targeting antibodies. Moreover, G36 inserted Tyr99 into the hydrophobic “Phe43 pocket” of gp120 to mimic the CD4 Phe43‐gp120 interaction (Figure 4G), similar to J3 which used Tyr99.^[^ ^19^, ^20^ ^]^ Mimicry of CD4 Phe43 was also observed in the human VRC01‐class antibodies, such as N6 and VRC‐PG20,^[^ ^3^, ^21^ ^]^ which utilized Tyr54 or Trp54 to interact with the gp120 pocket, demonstrating the ability of immune systems to take advantage of this site of vulnerability on HIV‐1 Env trimers. Overall, R27 and G36 bound at the CD4bs, in the canyon between two gp120 subunits with quaternary interactions, mimicking CD4 binding.

### 2.6 Ultrapotent and Broad HIV‐1 Neutralizing Bispecific Antibodies

**Figure 5:**
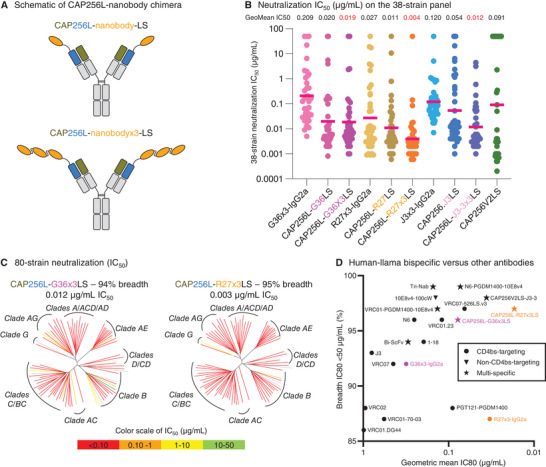
Ultra‐potent HIV‐1 bispecific antibodies from attaching nanobodies to the light chain of V2‐apex‐directed antibody CAP256V2LS. A) Schematic of CAP256L‐nanobody chimeras. B) 38‐strain neutralization of nanobodies. C) 80‐strain panel neutralization. Dendrograms display the diversity of tested viral strains, with branches colored according to neutralization potency (non‐neutralized branches shown in gray). D) Comparison of neutralization breadth and potency for R27 and G36 constructs with other potent antibodies on the 208‐strain panel. Data for CAP256L‐G36×3LS and CAP256L‐R27×3LS are estimated from 80‐strain data in panel C. Bispecific and trispecific antibodies are shown as stars.

To further improve the potency and breadth, we attached the two CD4bs‐targeting nanobodies, G36 and R27, to the N‐terminus of the light chain of a V2‐apex‐targeting broadly neutralizing antibody CAP256V2LS^[^ ^22^ ^]^ (**Figure** **5**A), creating human‐llama bispecific antibodies.^[^ ^6^ ^]^ A 38‐strain panel test showed that conjugating nanobody monomers (G36, R27, and J3) with CAP256V2LS improved neutralization potency over their triplet conjugation on human IgG1 Fc domain (x3‐IgG2a format described above) (Figure 5B). While CAP256L‐G36×3LS was not better than CAP256L‐G36LS, both CAP256L‐R27×3LS and CAP256L‐J3×3LS substantially improved neutralizing potency, with CAP256L‐R27×3LS being the most potent (geometric mean IC_50_ = 0.004 µg mL^−1^). We then tested CAP256L‐G36×3LS and CAP256L‐R27×3LS on an additional 42 strains. Data obtained from an overall 80‐strain panel showed that the two bispecific antibodies neutralized over 94% of cross‐clade HIV‐1 viruses ultrapotently, with geometric mean IC_50_s of 0.012 and 0.003 µg mL^−1^, respectively (Figure 5C, with explicit IC_50_ and IC_80_ values provided in Dataset S1, Supporting Information). Even though we did not test them on a larger panel, the 80‐strain panel data allowed us to make reliable estimations of the breadth and potency of the two bispecific antibodies on 208‐strain panel (Figure S9, Supporting Information).^[^ ^23^ ^]^ The estimated neutralization IC_80_ on a 208‐strain panel suggested the combined potency and breadth for CAP256L‐R27×3 to exceed other published HIV‐1 bNAbs, as well as previously reported multi‐specific antibodies (Figure 5D, with predicted IC_50_ and IC_80_ values provided in Dataset S1, Supporting Information).

### 2.7 Structural Basis for the Broad HIV‐1 Neutralization of CAP256L‐R27×3LS

**Figure 6:**
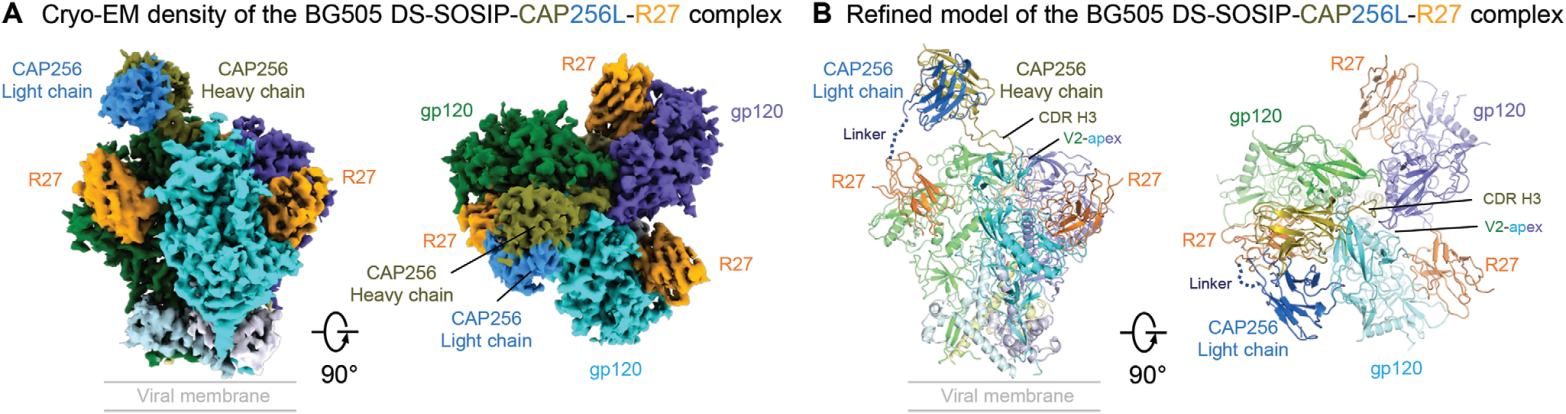
Cryo‐EM structure of CAP256L‐R27LS Fab in complex with HIV‐1 Env reveals CAP256 and R27 to bind prefusion‐closed trimer at V2‐apex and CD4bs simultaneously. Cryo‐EM density A) and refined model B) for the CAP256‐R27‐BG505 DS SOSIP complex were shown in two 90°‐views and colored by chains. The chimera antibody bound to the HIV‐1 Env with CAP256L CDR H3 (colored olive) inserted into the V2‐apex and the CAP256LS light chain (skyblue)‐linked R27 contacting one of the CD4‐binding sites, however, the density of the flexible linker between R27 and CAP256L light chain was disordered. Three copies of R27 were observed to bind to each of the 3 CD4bs on the HIV‐1 Env trimer, indicating the other two R27s were from different chimeric antibodies.

We were unable to obtain a cryo‐EM structure for CAP256L‐R27×3 bound to BG505 DS‐SOSIP due to antibody‐induced aggregation but did succeed in determining a cryo‐EM structure for CAP256L‐R27 bound to BG505 DS‐SOSIP. This structure revealed a single CAP256V2LS Fab to bind at the V2‐apex and three copies of R27 each to bind to a CD4bs of the trimer (**Figure** **6**; Figure S10, Table S1, Supporting Information). Even though the cryo‐EM density for the linker between R27 and CAP256 light chain was disordered, one of the bound R27s could be identified by its proximity to link with the light chain of CAP256 that bound to the V2‐apex (Figure 6), and the other two R27s were from separate CAP256L‐R27 molecules (Figure S10F, Supporting Information).

### 2.8 Autoreactivity and Enhanced Pharmacokinetics of CAP256L‐R27×3LS

**Figure 7:**
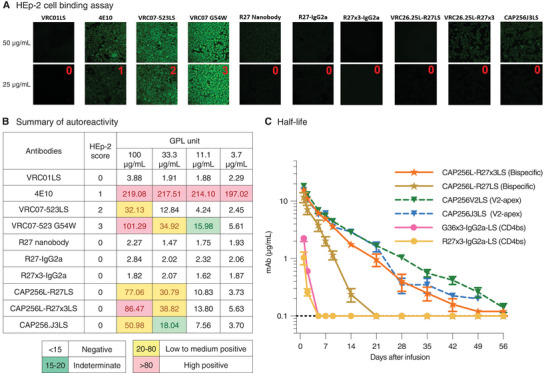
CAP256L‐R27×3LS autoreactivity and half‐life in human FcRn‐Fc KI mice. A) Autoreactivity of antibodies determined by HEp‐2 cell binding assay. B) Summary of autoreactivity of antibodies determined by HEp‐2 cell binding assay and anti‐cardiolipin ELISA assay. C) In vivo half‐life of CAP256L‐nanobody variants assessed in a human FcRn‐Fc knock‐in mouse model.

Finally, to evaluate whether the newly identified and engineered R27‐related nanobody molecules are suitable for clinical use, we sought to determine their autoreactivity and pharmacokinetics. While three control antibodies, 4E10, VRC07‐523LS, and VRC07‐G54W, showed binding scores of 1, 2, and 3, respectively, neither R27 nor its variants showed binding to Hep‐2 cells, indicating no antinuclear antibody (ANA) response (**Figure** **7**A). R27, R27‐IgG2a, and R27×3‐IgG2a showed no binding to cardiolipin. Only CAP256L‐R27LS and CAP256L‐R27×3LS showed low levels of binding to cardiolipin, which is similar to that of CAP256.J3LS (Figure 7B). Depending on administration methods, nanobody x3 was reported to have a very short half‐life in mice, ranging from 0.8 to 9.5 hours.^[^ ^24^ ^]^ Our pharmacokinetics analysis using human FcRn‐Fc‐KI mice revealed that although Fc conjugation can extend the half‐life of G36×3 and R27×3, they were cleared from mice within 5 days (Figure 7C). While monomeric R27 conjugated to CAP256 exhibited a bit longer but still less than optimal half‐life, the CAP256L‐R27×3LS half‐life was just a little bit shorter than the parent CAP256V2LS (Figure 7C).

## 3 Discussion

Multiple animal species have evolved alternatives to the heavy‐light antibody recognition utilized by the human immune system,^[^ ^5^, ^18^, ^25^ ^]^ and these alternatives provide unique ways to bypass HIV's Env defenses. Immunization in cows with prefusion‐closed Env trimer elicits broadly neutralizing antibodies, with cow‐specific D‐regions of up to 48 residues, extending from the body of the antibody to reach conserved elements of the CD4bs.^[^ ^25a^ ^]^ Immunization in llamas with gp140 elicited nanobodies such as J3, with VHH recognition enabled mimicry of CD4.^[^ ^5^, ^19^ ^]^ Serum neutralization however from the gp140 immunized llamas was not broadly neutralizing. Here we show how immunizations of a llama with a prefusion‐closed trimer initially yielded only autologous neutralization, but after repeated immunizations developed a broadly neutralizing response. Three doses of immunization with the same immunogen in humans yield autologous neutralizing antibodies that target the fusion‐peptide site of vulnerability.^[^ ^10^, ^11^ ^]^ Short‐term immunization of BG505 DS‐SOSIP seems to direct most of the antibody response to the glycan‐free base region, partially due to the inaccessibility of broadly neutralizing epitopes covered by surface glycans. It is possible that initially elicited, base‐targeting non‐neutralizing antibodies cover the highly immunogenic glycan‐free base, allowing antibody responses against neutralizing epitopes to develop after extended periods of immunizations. Indeed, broad serum neutralization responses were observed after 6 times of BG505 DS‐SOSIP immunization, which was still improving in terms of both breadth and potency when we terminated the study on day 271. More importantly, although the CD4bs may have been difficult to access due to their surrounding glycans, the elicited serum neutralization was mapped to be primarily VRC01‐like, and therefore CD4bs targeting.

We further identified CD4bs nanobodies with substantially higher potency than J3. While nanobody phage library screening using glycan base‐covered trimer only identified two neutralizing nanobodies, G36 and Day271_G1, screening with CD4bs‐specific subdomain probe RSC3 led to many neutralizing nanobodies exclusively targeting the CD4bs. CDR comparison and lineage analysis allowed us to identify nanobody candidates of interest from day 271 library based on neutralizing data of the nanobodies in the same lineage from the day 188 library. Although G36 and R27 both recognize CD4bs on HIV‐1 Env trimer, they demonstrated highly differential neutralization potency and breadth. The triplicate format (nanobody x3‐IgG2a) resulted in more potency improvement for R27 than for G36, which is likely due to their different binding pattern and approaching angles to the CD4bs. It is worth noting that the enhanced potency was not proportional to the number of CD4bs‐binding moieties in these molecules, suggesting a complex mechanism of neutralization. A combination of G36, R27, or J3, especially their triplicate format with the potent V2‐directed antibody, CAP256V2LS, yielded bispecifics with even greater neutralization potency.

Nanobody multimerization followed by bispecific conjugation with compatible human antibodies seems to be a generalizable strategy for nanobody function improvement. Of note, the newly engineered CAP256L‐J3‐3×3LS, a human‐llama bispecific antibody conjugating the triplicate of J3 variants J3‐3 to CAP256V2LS, exhibited more than 4 times improved potency than the previously reported bispecific antibody CAP256.J3LS. The best of the bispecifics from this study, CAP256L‐R27×3LS, was three times more potent than CAP256L‐J3‐3×3LS and reached levels of potency that rivaled the previously reported best multi‐specific antibodies developed thus far. CAP256L‐R27×3LS neutralized 93% of an 80‐strain panel with a geometric mean IC_80_ of 0.008 µg mL^−1^. Computational extrapolation predicted neutralization of the 208‐strain panel with a breadth of 97% and a geometric mean IC_80_ of 0.017 µg mL^−1^ (Figure S9A, Supporting Information). In comparison, the previously reported bispecific antibody with the best potency, CAP256V2LS‐J3‐3, neutralizes 98% of the 208‐strain panel with a geometric mean IC_80_ of 0.036 µg mL^−1^,^[^ ^6^ ^]^ and trispecific antibody N6‐PGDM1400‐10E8v4 neutralizes 99% with a geometric mean IC_80_ of 0.073 µg mL^−1^.^[^ ^7^ ^]^ We note that if VRC01 had comparable potency as CAP256L‐R27×3LS, the prevention efficacy would be expected to exceed 90% in the antibody‐mediated prevention (AMP) study.^[^ ^26^ ^]^

It is believed that low‐dose repeated mucosal challenge in macaques closely mimics clinical HIV‐1 infection scenarios.^[^ ^27^ ^]^ In one such study, a single injection of 10–1074, 3BNC117, or VRC01 protected macaques from SHIV_AD8_ infection until the median plasma concentrations declined to 0.17 – 1.83 µg mL^−1^, values that are comparable to their IC_80_s determined in vitro.^[^ ^28^ ^]^ If the observed correlation between virus breakthrough and in vitro IC_80_s of bNAbs holds true for different virus strains in humans, given its extremely low IC_80_, the protective serum level of CAP256L‐R27×3LS in humans could be at least 500‐fold below 10 µg mL^−1^. Importantly, CAP256L‐R27×3LS is not auto‐reactive at such a low concentration in our assay. Although CAP256L‐R27×3LS exhibited a slightly shorter half‐life than CAP256V2LS alone, its superior potency and breadth make it a promising bNAb for therapeutic and/or prophylactic application. Additional strategies such as reduction of net positive charge^[^ ^29^ ^]^ or through FC alterations, such as YTE (M252Y/S254T/T256E)^[^ ^30^ ^]^ or DHS (L309D/Q311H/N434S),^[^ ^31^ ^]^ may enable its half‐life to be further extended.

It remains to be seen if this bispecific can avoid anti‐antibody responses to enable its therapeutic and prophylactic use in humans. When used as an HIV‐1 therapeutic, the simultaneous targeting of V2‐apex and CD4bs on HIV‐1 Env trimers by CAP256L‐R27×3LS will likely result in less viral escape. In the future, it would also be interesting to see whether the glycan‐base‐covered BG505 DS‐SOSIP^[^ ^32^ ^]^ or combinations of heterologous base‐covered Env trimers can elicit broader neutralizing responses with less immunizations or over a shorter time period.

## 4 Experimental Section

### Expression and Purification of BG505 DS‐SOSIP and Related Proteins

BG505 DS‐SOSIP protein was expressed and purified as previously described.^[^ ^9^ ^]^ Glycan‐base (BG505 DS‐SOSIP.4mut_N502‐660) and CD4bs knockout (KO 4115) constructs of BG505 were expressed with an N‐terminal scFc tag, separated by an HRV‐3C cleavage site. The plasmids were transfected into Freestyle 293‐F cells (Thermo) and protein was expressed for 6 days at 37 °C. The cell supernatant was collected by centrifugation and applied to Protein A resin (Cytivia), after which the HIV Env was liberated by cleavage with HRV‐3C. The flowthrough was collected and applied to a Superdex S‐200 gel filtration column, after which the protein was concentrated to 1 mg mL^−1^, flash frozen with 10% glycerol, and stored at −80 °C until use. His‐tagged RSC3 was first purified from day‐5 supernatant of transiently transfected 293F cells using Ni‐NTA‐resin (Qiagen) and monomeric RSC3 was further purified through a HiLoad 16/600 Superdex200 sizing column (Cytiva) on an AKTA Pure FPLC system. HIV‐1 fusion peptide (FP8) was synthesized and biotinylated by GenScript.

### Llama Immunizations and Nanobody Phage Library Construction

Llama immunization procedures were performed by following Capralogics Inc. ACUC protocol. One llama (Capralogics) was immunized subcutaneously with 1 mg of recombinant BG505 DS‐SOSIP protein in the presence of Complete Freund's Adjuvant (CFA) at day 0, and boost immunized with 0.5 mg of BG505 DS‐SOSIP protein in the presence of Incomplete Freund's Adjuvant (IFA) on day 21, 42, 63, 84, 105, 126, 147, 168, 189, 210, 231, and 257, respectively. Test blood/serum samples were taken on days 0, 52, 73, 94, 115, 136, 157, 178, 199, 220, 241, and 271 for antibody‐antigen binding tests and neutralization tests. 300 mL of whole blood were collected on days 188 and 271, and peripheral blood mononuclear cells (PBMCs) were isolated and used for nanobody phage library construction as previously described.^[^ ^18^ ^]^

### Phage Screening for HIV‐1 Env Binding Nanobodies

Four HIV‐1 Env‐related proteins were used for phage screening: Env trimer (BG505 DS‐SOSIP), Glycan base trimer (BG505 DS‐SOSIP.4mut_N502‐660), RSC3 and biotinylated Fusion peptide. For Env trimer, glycan base trimer or RSC3 screening, three wells of MaxiSorp 96‐well plate (Thermo Fisher Scientific) were first coated with lectin (EMD Millipore, L8275, 100 µg mL^−1^ in PBS) at 4 °C overnight, washed with PBS with 0.1% Tween‐20 three times and blocked with 5% non‐fat milk in PBS at room temperature for 1 h, then coated with 50 µL of 100 µg mL^−1^ proteins at room temperature for 2 h. One well without target protein was included as a non‐coated control. For biotinylated FP8 screening, two wells of Streptavidin‐coated plate (Thermo Fisher Scientific) were coated with 50 µL of FP8 (100 µg mL^−1^ in PBS) at 4 °C overnight, washed and blocked with non‐fat milk. Another well with 50 µL of PBS was used as a non‐coated control. Nanobody phages were added to wells and screened as previously described.^[^ ^18^ ^]^

### Enzyme‐Linked Immunosorbent Assay

After one or two rounds of selection, TG‐1 cells from sub‐libraries were plated and colonies were picked to prepare periplasmic extracts containing crude nanobodies. Serum samples from different time points and nanobody candidates were tested for their binding to individual target proteins by enzyme‐linked immunosorbent assay (ELISA) as previously described.^[^ ^18^ ^]^ In brief, glycan proteins were coated onto Maxisorp plates indirectly through lectin, and biotinylated FP8 was coated to streptavidin‐coated plates. Plates were washed and blocked, and then 100 µL of diluted serum samples or undiluted nanobody‐containing supernatant were added and incubated for 2 h at room temperature. Horseradish peroxidase (HRP) conjugated goat anti‐alpaca VHH domain‐specific antibody (Jackson ImmunoResearch) and tetramethylbenzidine (TMB) (Thermo Fisher Scientific) were used for developing ELISA signals, which were measured with Synergy microplate reader (BioTek Gen5).

### Expression and Purification of Nanobodies and Fc Conjugated Nanobody Variants

Nanobodies were expressed and purified as previously described.^[^ ^18^ ^]^ In brief, phagemids of lead nanobodies were extracted from TG‐1 cells and transformed into WK6 cells (ATCC). WK6 cells were cultured in 2YT medium and induced by IPTG for nanobody expression. Cells were pelleted and treated with polymyxin B to release nanobodies from periplasmic region. Nanobodies in the supernatant were purified using a Capturem His‐tagged purification kit (Takara) or complete His‐tag purification resin (Roche), dialyzed, and filtered sterile before being used for downstream assays. Monomeric or multimeric nanobody sequences were fused to the Fc region of human IgG1 through llama IgG2a hinge and cloned into the pVRC8400 vector. In multimeric form, nanobody units were connected through (GGGGS)×3 flexible linkers. The Fc fusion constructs were expressed in Expi293 cells and antibodies in the supernatant were purified using protein A, concentrated, dialyzed, and filtered sterile.

### HIV Neutralization

Neutralization was measured using single‐round‐of‐infection HIV‐1 Env‐pseudoviruses and TZM‐bl target cells, as described previously.^[^ ^33^ ^]^ Neutralization curves were fit by nonlinear regression using a 5‐parameter hill slope equation. The neutralization titers were calculated as a reduction in luminescence units compared with control wells and reported as 50% or 80% inhibitory concentration (IC_50_ or IC_80_) in micrograms per milliliter.

### Biolayer Interferometry Assay for Epitope Mapping

A 96‐Channel Ultra High Throughput Octet RH96 System was used to map the binding epitopes of 6xHis‐tagged nanobodies. Assays were performed at 30 °C in tilted black 384‐well plates (Geiger Bio‐One) in PBS + 0.02% Tween20, 0.1% BSA, 0.05% sodium azide with agitation set to 1000 rpm. His‐tagged nanobodies (50 µg mL^−1^) were loaded onto Ni‐NTA biosensors for 150 seconds. Bindings to HIV‐1 Env were measured by dipping immobilized nanobodies into solutions of 200 nm BG505 DS‐SOSIP without or with 720 nm Fabs of antibodies with known epitopes for 180 seconds. Fabs of antibodies VRC01, VRC34.01, 1E6, PGT145 and 10–1074 were used for competing the CD4bs, FP, Env‐base, V2‐apex and glycan V3 epitopes, respectively. Parallel correction to subtract systematic baseline drift was carried out by subtracting the measurements recorded for a loaded sensor dipped into a buffer‐only control well. The degree of competition was calculated as (the ratio of responses between BG505 DS‐SOSIP in the presence of blocking Fab and BG505 DS‐SOSIP alone)×100% and defined as three categories: complete (<30%), partial (30–60%) and slight (60‐80%).

### Biolayer Interferometry Assay to Measure Nanobody Affinity

The BLI assay was performed using a Sartorius Octet R2 instrument to determine the affinity of BG505 DS‐SOSIP to nanobodies. In brief, 6×His‐tagged G36 and R27 were immobilized onto Ni‐NTA biosensors at 10 µg mL^−1^, and G36×3‐IgG2a and R27×3‐IgG2 were immobilized onto Protein A biosensors at 20 µg mL^−1^ for 60 seconds. Biosensors were then dipped into a solution containing BG505 DS‐SOSIP for 300 seconds followed by dissociation for 600 seconds. Sensorgrams of the concentration series were corrected with corresponding blank curves and fitted globally with Octet evaluation software using a 1:2 bivalent analyte model of binding.

### Cryo‐EM Data Collection, Processing, and Model Refinement

Cryo‐EM specimens of HIV‐1 Env trimer BG505 DS‐SOSIP complexes with nanobodies R27 and G36 were prepared by vitrification using a ThermoFisher Scientific Vitrobot Mark IV plunger. Quantifoil R 2/2 gold grids were glow‐discharged using a PELCO easiGlow glow‐discharger (air pressure: 0.39 mBar, current: 20 mA, duration: 30 s) immediately before use. Data was collected using SerialEM^[^ ^34^ ^]^ with a ThermoFisher Titan Krios G1 electron microscope equipped with a Gatan K2 Summit direct electron detector operating in the counting mode (Table S1, Supporting Information). For structure of CAP256L‐R27LS Fab in complex with BG505 DS‐SOSIP, Env was mixed with the CAP256L‐R27LS Fab at 1.0 to 1.2 molar ratio (protomer to Fab) at a final total protein concentration of ≈3–4 mg mL^−1^ and adjusted to have a final concentration of 0.005% (w/v) n‐Dodecyl β‐D‐maltoside (DDM) to prevent preferred orientation and aggregation during vitrification. Cryo‐EM grids were prepared by applying 3 µL of sample to a fresh glow‐discharged carbon‐coated copper grid (CF 1.2/1.3 300 mesh). The sample was vitrified in liquid ethane using a Vitrobot Mark IV with a wait time of 30 s, a blot time of 3 s, and a blot force of 0. Cryo‐EM data were collected on a Titan Krios operating at 300 keV, equipped with a K3 detector (Gatan) operating in counting mode. Data were acquired using Leginon.^[^ ^35^ ^]^ The dose was fractionated over 50 raw frames.

Cryo‐EM data process workflow for the BG505 DS‐SOSIP in complex with llama nanobodies and the bi‐specific antibody, including motion correction, CTF estimation, particle picking and extraction, 2D classification, ab initio reconstruction, heterogeneous refinement, homogeneous refinement, non‐uniform refinement, and local resolution estimation, were carried out in cryoSPARC 3.3.^[^ ^36^ ^]^ For the three complexes, 315969, 414002, and 236154 particles, respectively, were selected after 3D Ab‐Initio classification and heterogeneous refinement for further refinement, and final cryo‐EM density maps after sequential homogeneous and non‐uniform refinements were used for iterative manual model building and real‐space refinement in Coot^[^ ^37^ ^]^ and in Phenix.^[^ ^38^ ^]^ The coordinates of cryo‐EM structures J3‐BG505 DS‐SOSIP (PDB ID: 7LPN) and CAP256V2LS‐J3‐3‐ BG505 DS‐SOSIP (PDB. ID: 8FIS) were used as initial models. Molprobity^[^ ^39^ ^]^ was used to validate geometry and check structure quality at each iteration step. UCSF Chimera and ChimeraX^[^ ^40^ ^]^ were used for map fitting and manipulation.

### Antibody‐Env interface Analysis

The buried interface areas, hydrogen bonds, and salt bridges between the bound antibodies and HIV‐1 Env were analyzed using the PDBePISA website (https://www.ebi.ac.uk/pdbe/pisa/pistart.html).^[^ ^41^ ^]^

### Neutralization Fingerprint Analysis

The neutralization fingerprints of day 188 and day 271 serum samples, defined as the potency pattern with which the sera neutralized a set of 60 HIV‐1 strains, were analyzed and compared as described previously.^[^ ^14^ ^]^

### Nanobody Lineage Analysis

Nucleotide sequences were submitted to IMGT Vquest server to assign the germline gene (https://www.imgt.org/IMGT_vquest/input). The phylogenetic trees were prepared by gctree with default parameters.^[^ ^42^ ^]^

### Pharmacokinetic Study in Human Neonatal Fc receptor‐Fc (FcRn‐Fc) Transgenic Mice

Human FcRn‐Fc transgenic mice (FcRn‐/‐ hFcRn‐Fc Tg mice, JAX stock # 029686, The Jackson Laboratory) were used to assess the pharmacokinetics of selected antibodies. Each animal was infused intravenously with 5 mg mAb/kg of body weight. Whole blood samples were collected on days 1, 2, 5, 7, 9, 14, 21, 28, 35, 42, 49, and 56. Serum mAb levels were measured by ELISA using an anti‐idiotypic antibody to CAP256 or BG505 DS‐SOSIP trimer as described previously.^[^ ^43^ ^]^ All mice were bred and maintained under pathogen‐free conditions at the American Association for the Accreditation of Laboratory Animal Care‐accredited Animal Facility at the National Institute of Allergy and Infectious Diseases and housed in accordance with the procedures outlined in the Guide for the Care and Use of Laboratory Animals. All the mice were between 6 and 13 weeks of age. The study protocol was evaluated and approved by the National Institutes of Health's Animal Care and Use Committee (ASP VRC‐20‐893).

### Predicting Nanobody Neutralization Against 208 HIV‐1 Isolates

Using the measured neutralization for bispecific antibodies against 80 HIV‐1 isolates, it was extrapolated how these antibodies‐of‐interest would neutralize a larger panel of 208 isolates. Briefly, the measurements were combined with neutralization from 80 reference monoclonal antibodies measured against the 208 isolates. Then matrix completion was applied via nuclear norm minimization, which found linear combinations of the reference antibodies that matched the neutralization from the antibodies of interest and used these linear combinations to infer the remaining measurements.^[^ ^23^ ^]^ To assess prediction error, 10% of available measurements and applied matrix completion were withheld to compare the predicted‐versus‐measured values. The error of these predictions was 4‐fold (i.e., a predicted IC_80_ = 0.4 µg mL^−1^ should lie between 0.4/4 = 0.1 and 0.4×4 = 1.6 µg mL^−1^), far smaller than 10^7^‐fold range of the neutralization data. As done previously, IC_80_s were inverted and logged (IC_80_→ log_10_[1/IC_80_]) prior to matrix completion and then reverted after matrix completion.^[^ ^23^ ^]^ Inverting ensures that the smallest values representing the strongest responses were predicted more accurately than weak responses. Log transformation prevents strong neutralization measurements from overpowering the predictions. Each reference antibody had at least 40 concrete measurements against the 80‐isolate panel (i.e., ignoring bounded values such as IC_80_>100 µg mL^−1^) to enable comparison with the antibodies of interest. For each antibody of interest, the fraction of the 208‐isolate panel was isolated which it neutralized with an IC_80_<50 µg mL^−1^. To account for the 4‐fold prediction error, several thousand simulations were run, where every predicted IC_80_ was multiplied by a factor between ¼ and 4 (equally sampled in a log‐scale), the number of IC_80_s<50 µg mL^−1^ was counted, and fit the resulting distribution.

### Autoreactivity

The autoreactivity of the antibodies was assessed using the ANA Hep‐2 Test System (ZEUS Scientific, Cat. No: FA2400EB) and anticardiolipin ELISA kit (Inova Diagnostics Cat. No.: 708625). Briefly, to evaluate the binding of the antibodies to Hep‐2 cells, all the antibodies were tested at 25 and 50 µg mL^−1^ in PBS buffer following the instructions from the manufacturer of the ANA Hep‐2 Test System. The VRC01LS, 4E10, VRC07‐523‐LS, and VRC07‐G54W antibodies were used as controls. Slides were imaged on a Nikon Eclipse Ts2R microscope with a 20× objective lens for 500 ms. The fluorescent signals of the control antibodies at 25 µg mL^−1^ were scored as 0, 1, 2, and 3, respectively. The fluorescent signals of the test antibodies were estimated visually in comparison to the control ones. Scores over 1 at 25 µg mL^−1^ were defined as autoreactive and between 0 and 1 as mildly autoreactive. For the cardiolipin ELISA, all the antibodies were tested at 100 µg mL^−1^, followed by a 3‐fold serial dilution. IgG phospholipid (GPL) units were derived from the standard curve. A GPL score below 20 was considered as not reactive, between 20 and 80 as a low positive, and greater than 80 as a high positive. The reported results were representative of two independent experiments.

### Data Presentation

Figures were arranged in PowerPoint.

### Statistical Analysis

Statistical analysis was performed with GraphPad Prism 9 software. Neutralization titers of individual strains were calculated by fitting nonlinear regression using a five‐parameter hill slope equation, and the panels IC_50_ and IC_80_ were presented as geometric mean. The exact sample size for each experimental group was reported in figure legend and/or shown in figure as the number of branches or dots. The *p*‐value < 0.05 was considered to be significant.

## Conflict of Interest

The National Institutes of Health was in the process of filing a patent application in connection with this work on which J.X., T.Z., B.Z., A.F.N., A.P., C.S., Y.D.K, A.S.O., E.S.Y., N.A.D., R. Casellas, and P.D.K. were contributors. Other authors declare no competing interests.

## Author Contributions

J.X. and T.Z. contributed equally to this work. J.X., T.Z., R. Casellas., and P.D.K conceptualized the project. J.X. led the llama immunization, nanobody identification, production, and engineering studies. T.Z. led the epitope mapping and cryo‐EM studies. K.M., S.O., and E.T. performed neutralization analysis. B.Z. engineered and produced bispecific antibodies. C.L. and J.M. performed autoreactivity analysis. A.F.N. performed epitope mapping analysis. A.P. led the pharmacokinetic analysis. C.S. performed nanobody lineage analysis. M.F.B. and Y.D.K. produced antibodies for pharmacokinetic analysis. A.C., R. Chaudhary, X.C., A.S.O., and I‐T.T. provided critical reagents. T.E. performed the 208‐strain neutralization estimation analysis. B.C.L. and M.K.L. performed a 208‐strain neutralization analysis. J.E.B., N.C.M., R.S.R., and T.S. collected cryo‐EM data. R.R. performed a neutralization fingerprinting analysis. P.C. produced nanobodies and performed kinetics analysis. S.W. assisted with data visualization. E.S.Y. performed the pharmacokinetic analysis. Y.T., L.S. led the cryo‐EM data collection. N.A.D. led the neutralization analysis. J.X., T.Z., S.W., and P.D.K. wrote the original draft of the paper, and all authors reviewed and approved the final manuscript.

## Supporting information

Supporting Information

Supplemental Table 1

## Data Availability

The data that support the findings of this study are openly available in the Electron Microscopy Data Bank and Protein Data Bank under entry IDs EMD‐41415, EMD‐41416, EMD‐41417, and PDB IDs 8TNG, 8TNH, 8TNI, respectively.
