# Supplementary material for: Ultrapotent Broadly Neutralizing Human‐llama Bispecific Antibodies against HIV‐1
Source: Adv Sci (Weinh). 2024 May 5;11(26):2309268. doi: 10.1002/advs.202309268 (PMC11234422; doi:10.1002/advs.202309268)
Supplement: Supplementary file 1 — Supporting Information [file ADVS-11-2309268-s002.pdf]

ADVANCED SCIENCE
Open Access

Supporting Information

for *Adv. Sci.*, DOI 10.1002/advs.202309268

Ultrapotent Broadly Neutralizing Human-llama Bispecific Antibodies against HIV-1

Jianliang Xu, Tongqing Zhou, Krisha McKee, Baoshan Zhang, Cuiping Liu, Alexandra F. Nazzari, Amarendra Pegu, Chen-Hsiang Shen, Jordan E. Becker, Michael F. Bender, Payton Chan, Anita Changela, Ridhi Chaudhary, Xuejun Chen, Tal Einav, Young Do Kwon, Bob C. Lin, Mark K. Louder, Jonah S. Merriam, Nicholas C. Morano, Sijy O'Dell, Adam S. Olia, Reda Rawi, Ryan S. Roark, Tyler Stephens, I-Ting Teng, Emily Tourtellott-Fogt, Shuishu Wang, Eun Sung Yang, Lawrence Shapiro, Yaroslav Tsybovsky, Nicole A. Doria-Rose, Rafael Casellas and Peter D. Kwong**

A Sera 60-strain neutralization (ID_{50})

	D188 serum	D271 serum
# Viruses	60	60
Total VS Neutralized		
ID50 >1:20	46	53
ID50 >1:100	36	45
ID50 >1:1000	13	31
ID50 >1:10000	0	5
% VS Neutralized		
ID50 >1:20	77	88
ID50 >1:100	60	75
ID50 >1:1000	22	52
ID50 >1:10000	0	8
Median ID50	359.0	1330.0
Geometric Mean	357.4	1031.9

B 60-strain neutralization fingerprinting

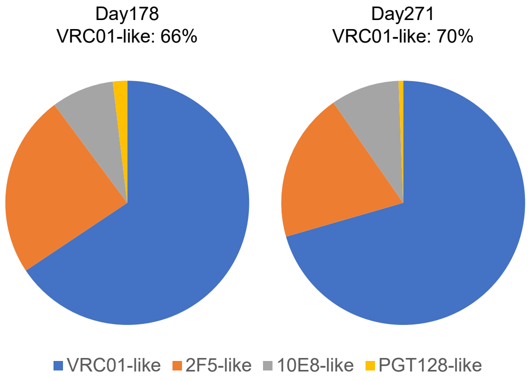

Figure S1. Serum neutralization response in BG505 DS-SOSIP immunized llama. (A) 60-strain serum neutralization. (B) Neutralization fingerprinting of day 188 and day 271 serum samples.

A Epitope mapping of 16 nanobodies from day 188 library

Nanobodies	Phage screening probe	% VRC01	% 1E6	% VRC34.01	Epitope
R11	RSC3	-10	-	-	CD4bs
R18	RSC3	-4	-	-	CD4bs
R21	RSC3	-4	-	-	CD4bs
R25	RSC3	-3	-	-	CD4bs
G36	Glycan base trimer	9	-	-	CD4bs
G42	Glycan base trimer	19	-	-	CD4bs
F7	FP8	-	-	-4	FP
G16	Glycan base trimer	-	-	14	FP
G25	Glycan base trimer	-	-	2	FP
G39	Glycan base trimer	-	-	9	FP
G40	Glycan base trimer	-	-	50	FP
G26	Glycan base trimer	-	5	2	Base & FP
G18	Glycan base trimer	-	6	-	Base
G41	Glycan base trimer	-	19	-	Base
T13	Env Trimer	-	21		Base
T52	Env Trimer	-	41	-	Base
% competition = binding (BG505 with Fab) / binding BG505 alone *100					

B Summary of 151 nanobodies from day 188 library

151 unique nanobodies (Day188)	Epitope mapping with Fabs					
	VRC01	VRC34.01	1E6	PGT145	10-1074	unaccounted
	CD4bs	FP	Base	V2	V3	
Env Trimer		4	19			46
Fusion Peptide		7	2			
Glycan base Trimer	2	17	26			
RSC3	28					

C Neutralization of 24 non-CD4bs targeting nanobodies (IC_{50} μg/mL)

		FP						Base/FP	Base			Unaccounted													
Clade	Virus	F7	G25	G39	G41	G40	G16	G26	E52	G18	E13	E2	E17	E23	E27	E30	E36	E40	E49	E62	E63	E64	E65	E66	E72
A	BG505.W6M.C2.T332N.SG3	>10	>10	>10	>10	>10	>10	>10	>10	>10	>10	>10	>10	>10	>10	>10	>10	>10	>10	0.262	>10	>10	>10	>10	>10
A	Q23.17.SG3	>10	>10	>10	>10	>10	>10	>10	>10	>10	>10	>10	>10	>10	>10	>10	>10	>10	>10	>10	>10	>10	>10	>10	>10
A	RW020.2.SG3	>10	>10	>10	>10	>10	>10	>10	>10	>10	>10	>10	>10	>10	>10	>10	>10	>10	>10	>10	>10	>10	>10	>10	>10
AC	3301.v1.c24.SG3	>10	>10	>10	>10	>10	>10	>10	>10	>10	>10	>10	>10	>10	>10	>10	>10	>10	>10	>10	>10	>10	>10	>10	>10
AD	Q168.a2.SG3	>10	>10	>10	>10	>10	>10	>10	>10	>10	>10	>10	>10	>10	>10	>10	>10	>10	>10	>10	>10	>10	>10	>10	>10
B	JRCSF.JB.SG3	>10	>10	>10	>10	>10	>10	>10	>10	>10	>10	>10	>10	>10	>10	>10	>10	>10	>10	>10	>10	>10	>10	>10	>10
B	QH0515.01.SG3	>10	>10	>10	>10	>10	>10	>10	>10	>10	>10	>10	>10	>10	>10	>10	>10	>10	>10	>10	>10	>10	>10	>10	>10
B	RHPA.7.SG3	>10	>10	>10	>10	>10	>10	>10	>10	>10	>10	>10	>10	>10	>10	>10	>10	>10	>10	>10	>10	>10	>10	>10	>10
B	TRO.11.SG3	>10	>10	>10	>10	>10	>10	>10	>10	>10	>10	>10	>10	>10	>10	>10	>10	>10	>10	0.639	>10	>10	>10	>10	>10
C	CAP256.206.C9.SG3	>10	>10	>10	>10	>10	>10	>10	>10	>10	>10	>10	>10	>10	>10	>10	>10	>10	>10	>10	>10	>10	>10	>10	>10
C	MW965.26.SG3	>10	>10	>10	>10	>10	>10	>10	>10	>10	>10	>10	>10	>10	>10	>10	>10	>10	>10	>10	>10	>10	>10	>10	>10
C	ZM233.6.SG3	>10	>10	>10	>10	>10	>10	>10	>10	>10	>10	>10	>10	>10	>10	>10	>10	>10	>10	>10	>10	>10	>10	>10	>10
C	ZM249.1.SG3	>10	>10	>10	>10	>10	>10	>10	>10	>10	>10	>10	>10	>10	>10	>10	>10	>10	>10	>10	>10	>10	>10	>10	>10
D	NKU3006.ec1.SG3	>10	>10	>10	>10	>10	>10	>10	>10	>10	>10	>10	>10	>10	>10	>10	>10	>10	>10	>10	>10	>10	>10	>10	>10
SIV	SIVmac251.30.SG3	>10	>10	>10	>10	>10	>10	>10	>10	>10	>10	>10	>10	>10	>10	>10	>10	>10	>10	>10	>10	>10	>10	>10	>10

D 14-strain neutralization of 6 CD4bs-targeting nanobodies from day 188 library (IC_{50} μg/mL)

Clade	Virus	G36	G42	R11	R18	R21	R25	J3
A	BG505.W6M.C2.T332N.SG3	0.007	0.006	0.006	0.003	0.005	0.0050	0.463
A	Q23.17.SG3	0.004	0.118	0.035	0.032	0.023	0.013	4.690
A	RW020.2.SG3	0.019	0.093	2.370	0.013	0.695	1.660	0.182
AC	3301.v1.c24.SG3	0.352	>10	0.966	>10	3.410	2.480	0.492
AD	Q168.a2.SG3	0.028	0.750	0.016	0.416	0.024	0.010	0.342
B	JRCSF.JB.SG3	0.015	>10	0.555	9.630	0.432	0.085	0.119
B	QH0515.01.SG3	0.052	1.760	0.595	0.463	0.091	0.038	0.901
B	RHPA.7.SG3	0.777	6.530	0.010	>10	0.019	0.033	0.173
B	TRO.11.SG3	9.040	0.554	0.959	0.404	1.130	2.450	1.840
C	CAP256.206.C9.SG3	0.421	>10	0.310	>10	0.155	0.100	0.596
C	MW965.26.SG3	0.388	>10	>10	>10	>10	1.640	0.008
C	ZM233.6.SG3	0.052	>10	>10	>10	>10	>10	4.100
C	ZM249.1.SG3	0.626	>10	>10	>10	>10	3.490	0.737
D	NKU3006.ec1.SG3	0.011	0.094	0.024	0.069	0.017	0.017	0.222
SIV	SIVmac251.30.SG3	>10	>10	>10	>10	>10	>10	>10

<0.01 0.01-0.1 0.1-1 1-10

Figure S2. Screening of HIV-1 neutralizing nanobodies from day 188 library. (A) Epitope mapping of 16 nanobodies from day 188 library. (B) Summary of epitope mapping of 151 nanobodies. (C) Neutralization of 24 non-CD4bs targeting nanobodies. (D) 14-strain neutralization of 6 CD4bs-targeting nanobodies from the day 188 library.

A Alignment of 6 CD4bs-targeting nanobodies (day188) with 17 selected nanobodies from day 271

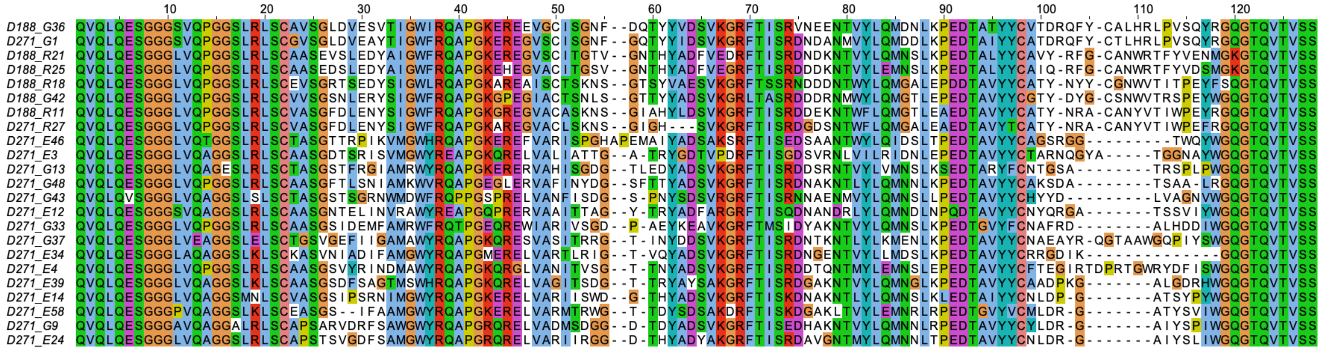

B 5-strain neutralization of 17 nanobodies from day 271 library (IC_{50} µg/mL)

Clade	Virus	R27	E3	E4	E12	E14	E24	E34	E39	E46	E58	G1	G9	G13	G33	G37	G43	G48	J3
A	BG505.W6M.C2.T332N.SG3	0.001	>10	>10	>10	>10	>10	>10	>10	0.501	>10	0.014	>10	>10	>10	>10	>10	>10	0.684
A	Q23.17.SG3	0.010	>10	>10	>10	>10	>10	>10	>10	2.470	>10	0.051	>10	>10	>10	>10	>10	>10	4.260
B	JRCSF.JB.SG3	0.524	>10	>10	>10	>10	>10	>10	>10	>10	>10	0.035	>10	>10	>10	>10	>10	>10	0.118
C	CAP256.206.C9.SG3	0.555	>10	>10	>10	>10	>10	>10	>10	>10	>10	>10	>10	>10	>10	>10	>10	>10	0.508
C	ZM233.6.SG3	1.420	>10	>10	>10	>10	>10	>10	>10	>10	>10	0.992	>10	>10	>10	>10	>10	>10	3.030

<0.01 | 0.01-0.1 | 0.1-1 | 1-10

C 10-strain neutralization of 42 nanobodies from 3 lineages (IC_{50} µg/mL)

Clade	Virus	D188_R1	D188_R3	D188_R4	D188_R5	D188_R7	D188_R8	D188_R9	D188_R10	D188_R11	D188_R13	D188_R14	D188_R16	D188_R17	D188_R19
A	BG505.W6M.C2.T332N.SG3	0.007	0.001	0.003	0.001	0.007	0.006	0.007	0.004	0.001	0.002	0.025	0.009	0.001	0.008
A	Q23.17.SG3	0.058	0.003	0.016	0.006	0.056	0.066	0.093	0.024	0.005	0.003	0.148	0.073	0.003	0.069
AE	C1080.c3.SG3	9.050	>10	3.400	>10	3.320	>10	>10	5.530	>10	>10	>10	>10	>10	>10
AG	269-12.SG3	>10	>10	>10	>10	>10	>10	>10	>10	>10	>10	>10	>10	>10	>10
B	JRCSF.JB.SG3	>10	0.196	0.786	0.407	>10	>10	8.190	>10	0.367	0.033	>10	8.66	0.117	>10
B	QH0515.01.SG3	0.353	0.043	0.055	0.068	0.429	0.507	0.632	0.336	0.403	0.027	0.912	0.197	0.040	0.747
C	CAP256.206.C9.SG3	>10	0.105	3.760	0.092	>10	>10	>10	>10	0.179	0.062	>10	>10	0.081	>10
C	CAP45.G3.SG3	>10	2.620	3.130	>10	>10	>10	>10	7.380	>10	>10	>10	>10	>10	>10
C	ZM233.6.SG3	6.750	>10	2.300	>10	8.830	4.260	8.800	2.130	>10	>10	>10	2.020	>10	3.740
D	A03349M1.vrc4a.SG3	1.530	1.460	1.250	>10	1.600	2.150	4.100	1.290	2.110	>10	2.370	1.940	>10	1.460

<0.001 | 0.001-0.01 | 0.01-0.1 | 0.1-1 | 1-10

Clade	Virus	D188_R20	D188_R21	D188_R23	D188_R24	D188_R25	D188_R26	D188_R27	D188_R28	D188_G36	D271_R1	D271_R3	D271_R7	D271_R8	D271_R20
A	BG505.W6M.C2.T332N.SG3	0.016	0.002	0.011	0.0009	0.001	0.002	0.0009	0.006	0.003	0.001	0.005	0.001	0.007	0.0006
A	Q23.17.SG3	0.062	0.008	0.051	0.001	0.002	0.002	0.004	0.036	0.0008	0.008	0.031	0.001	0.057	0.002
AE	C1080.c3.SG3	>10	>10	4.930	9.140	>10	>10	>10	2.440	0.579	>10	>10	>10	>10	>10
AG	269-12.SG3	>10	>10	>10	>10	>10	>10	>10	>10	6.320	>10	>10	>10	>10	>10
B	JRCSF.JB.SG3	>10	0.195	>10	0.045	0.041	0.241	0.161	>10	0.003	0.036	3.380	0.005	>10	0.075
B	QH0515.01.SG3	0.319	0.058	0.280	0.003	0.020	0.040	0.059	0.411	0.027	0.114	0.626	0.003	1.030	0.021
C	CAP256.206.C9.SG3	>10	0.117	>10	0.054	0.073	0.080	0.057	>10	0.291	0.035	1.590	0.047	>10	0.055
C	CAP45.G3.SG3	5.140	>10	>10	2.220	>10	3.650	>10	4.270	>10	>10	>10	2.590	>10	5.250
C	ZM233.6.SG3	2.990	>10	>10	3.850	>10	>10	>10	1.900	0.052	7.640	>10	1.840	8.050	0.979
D	A03349M1.vrc4a.SG3	1.180	>10	1.660	>10	>10	4.690	>10	1.480	1.500	>10	1.540	1.940	1.710	>10

Clade	Virus	D271_R25	D271_R27	D271_R30	D271_R35	D271_R38	D271_R42	D271_R46	D271_R47	D271_R49	D271_R51	D271_R54	D271_R55	D271_R59	D271_G1
A	BG505.W6M.C2.T332N.SG3	0.002	0.0008	0.0007	0.004	0.002	0.002	0.002	0.001	0.008	0.010	0.002	0.0009	0.001	0.017
A	Q23.17.SG3	0.041	0.005	0.002	0.062	0.004	0.003	0.009	0.003	0.072	0.065	0.021	0.002	0.003	0.036
AE	C1080.c3.SG3	>10	2.750	>10	9.860	>10	>10	1.600	1.920	6.540	>10	2.810	>10	1.440	>10
AG	269-12.SG3	>10	>10	>10	>10	>10	>10	>10	>10	>10	>10	>10	>10	>10	2.800
B	JRCSF.JB.SG3	8.640	0.317	0.005	1.590	0.024	0.015	0.740	0.072	>10	>10	4.240	0.044	0.103	0.048
B	QH0515.01.SG3	0.238	0.049	0.006	1.040	0.033	0.013	0.047	0.052	0.588	0.326	0.206	0.116	0.033	2.290
C	CAP256.206.C9.SG3	>10	0.635	0.052	>10	0.071	0.109	3.520	0.343	1.350	7.400	>10	0.034	0.257	>10
C	CAP45.G3.SG3	>10	1.540	4.570	>10	>10	1.120	1.360	1.740	>10	>10	>10	>10	2.980	>10
C	ZM233.6.SG3	4.480	1.320	6.060	4.570	>10	2.740	1.380	1.510	1.380	1.150	1.330	1.950	1.720	0.999
D	A03349M1.vrc4a.SG3	1.300	0.420	1.750	1.080	>10	1.930	0.480	0.541	0.907	1.060	0.910	9.610	0.593	>10

Figure S3. Screening of HIV-1 neutralizing nanobodies from day 271 library. (A) Alignment of day 271 nanobodies with selected day 181 nanobodies. (B) Neutralization of 17 unique nanobodies identified from the day 271 library. (C) 10-strain neutralization of 42 nanobodies from the three selected lineages. Names of nanobodies with over 90% breadth (IC_{50} <50 µg/mL) were highlighted in red.

A Nanobody formats

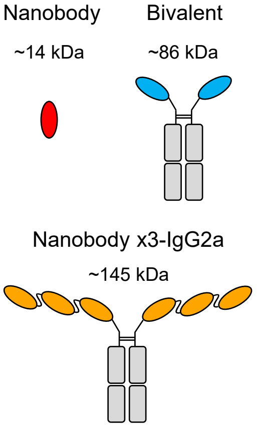

B 5-strain neutralization of engineered nanobodies (IC_{50} µg/mL)

Clade	Virus	G36 Nanobody	G36 Bivalent	G36 Nanobody x3-IgG2a	R27 Nanobody	R27 Bivalent	R27 Nanobody x3-IgG2a
A	BG505.W6M.C2.T332N.SG3	0.007	0.024	0.014	0.001	0.032	<0.01
A	Q23.17.SG3	0.004	0.015	0.012	0.010	0.047	<0.01
B	JRCSF.JB.SG3	0.015	0.035	0.022	0.524	0.043	<0.01
C	CAP256.206.C9.SG3	0.421	0.356	0.350	0.555	0.780	0.072
C	ZM233.6.SG3	0.052	0.205	0.247	1.420	0.170	0.041

<0.01 0.01-0.1 0.1-1 1-10

C 5-strain neutralization of engineered nanobodies (IC_{50} nM)

Clade	Virus	G36 Nanobody	G36 Bivalent	G36 Nanobody x3-IgG2a	R27 Nanobody	R27 Bivalent	R27 Nanobody x3-IgG2a
A	BG505.W6M.C2.T332N.SG3	0.479	0.272	0.095	0.073	0.372	<0.07
A	Q23.17.SG3	0.274	0.170	0.081	0.729	0.546	<0.07
B	JRCSF.JB.SG3	1.027	0.397	0.149	38.248	0.500	<0.07
C	CAP256.206.C9.SG3	28.835	4.045	2.381	40.510	9.069	0.072
C	ZM233.6.SG3	3.561	2.329	1.680	103.649	1.976	0.476

<0.07 0.07-0.1 0.1-1 1-10 10-50 >100

D 5-strain neutralization of engineered nanobodies (IC_{50} µg/mL)

Clade	Virus	D188_R11 Nanobody	D188_R11 Bivalent	D188_R11 Nanobody x3-IgG2a	D188_R18 Nanobody	D188_R18 Bivalent	D188_R18 Nanobody x3-IgG2a	D188_R25 Nanobody	D188_R25 Bivalent	D188_R25 Nanobody x3-IgG2a	D188_G42 Nanobody	D188_G42 Bivalent	D188_G42 Nanobody x3-IgG2a
A	BG505.W6M.C2.T332N.SG3	0.006	0.035	<0.01	0.003	0.011	<0.01	0.005	0.048	0.020	0.006	0.030	<0.01
A	Q23.17.SG3	0.035	0.033	<0.01	0.032	<0.01	<0.01	0.013	0.036	0.022	0.118	0.022	<0.01
B	JRCSF.JB.SG3	0.555	0.167	0.037	9.630	0.186	0.074	0.085	0.058	0.030	>10	0.603	0.124
C	CAP256.206.C9.SG3	0.310	1.820	0.271	>10	1.170	0.703	0.100	0.614	0.207	>10	4.750	1.250
C	ZM233.6.SG3	>10	0.853	0.144	>10	>10	7.040	>10	3.400	1.340	>10	>10	>10

Clade	Virus	D271_G1 Nanobody	D271_G1 Bivalent	D271_G1 Nanobody x3-IgG2a	D188_F7 Nanobody	D188_F7 Bivalent	D188_F7 Nanobody x3-IgG2a	D188_G25 Nanobody	D188_G25 Bivalent	D188_G25 Nanobody x3-IgG2a	D188_G39 Nanobody	D188_G39 Bivalent	D188_G39 Nanobody x3-IgG2a
A	BG505.W6M.C2.T332N.SG3	0.014	0.027	<0.01	>10	1.660	0.422	>10	>10	0.215	>10	2.850	0.586
A	Q23.17.SG3	0.051	0.027	<0.01	>10	>10	>10	>10	>10	2.040	>10	>10	>10
B	JRCSF.JB.SG3	0.035	0.046	<0.01	>10	>10	>10	>10	>10	1.700	>10	>10	>10
C	CAP256.206.C9.SG3	>10	1.490	0.331	>10	>10	>10	>10	>10	>10	>10	>10	>10
C	ZM233.6.SG3	0.992	0.138	0.069	>10	>10	>10	>10	>10	>10	>10	>10	>10

E 208-strain neutralization (IC_{80} µg/mL)

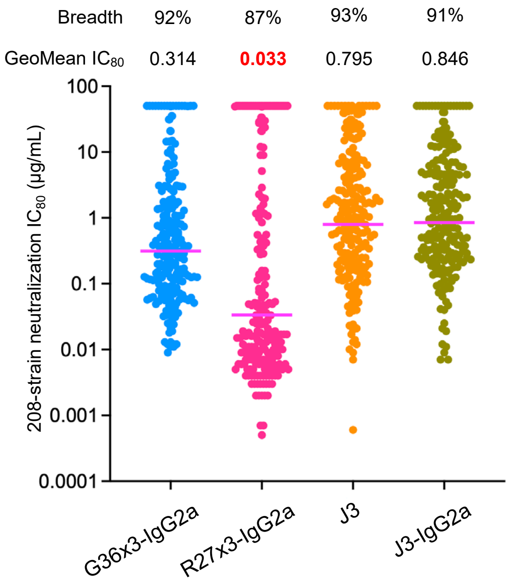

F Affinity of BG505 DS-SOSIP to nanobodies

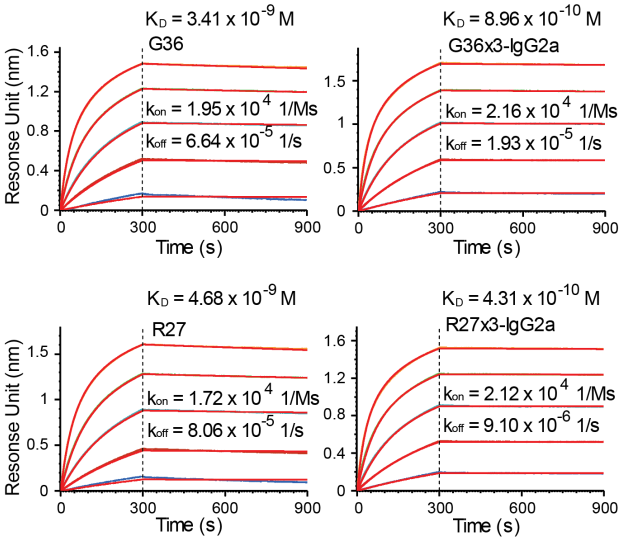

Figure S4. Improved neutralization potency and breadth by Fc conjugation and multimerization. (A) Scheme of nanobody forms. (B and C) 5-strain neutralization of nanobody monomers, bivalent and nanobody x3-IgG2a. (D) 5-strain neutralization of nanobody monomers, bivalent and nanobody x3-IgG2a. (E) IC_{80} of 208-strain neutralization. (F) Biolayer interferometry (BLI) analysis of different concentrations (25 nM, 50 nM, 100 nM, 200 nM, 400 nM) of BG505 DS-SOSIP binding to immobilized nanobody molecules. Red trace represents the kinetic fit. Raw data is shown underneath. Equilibrium (K_D) constants are provided.

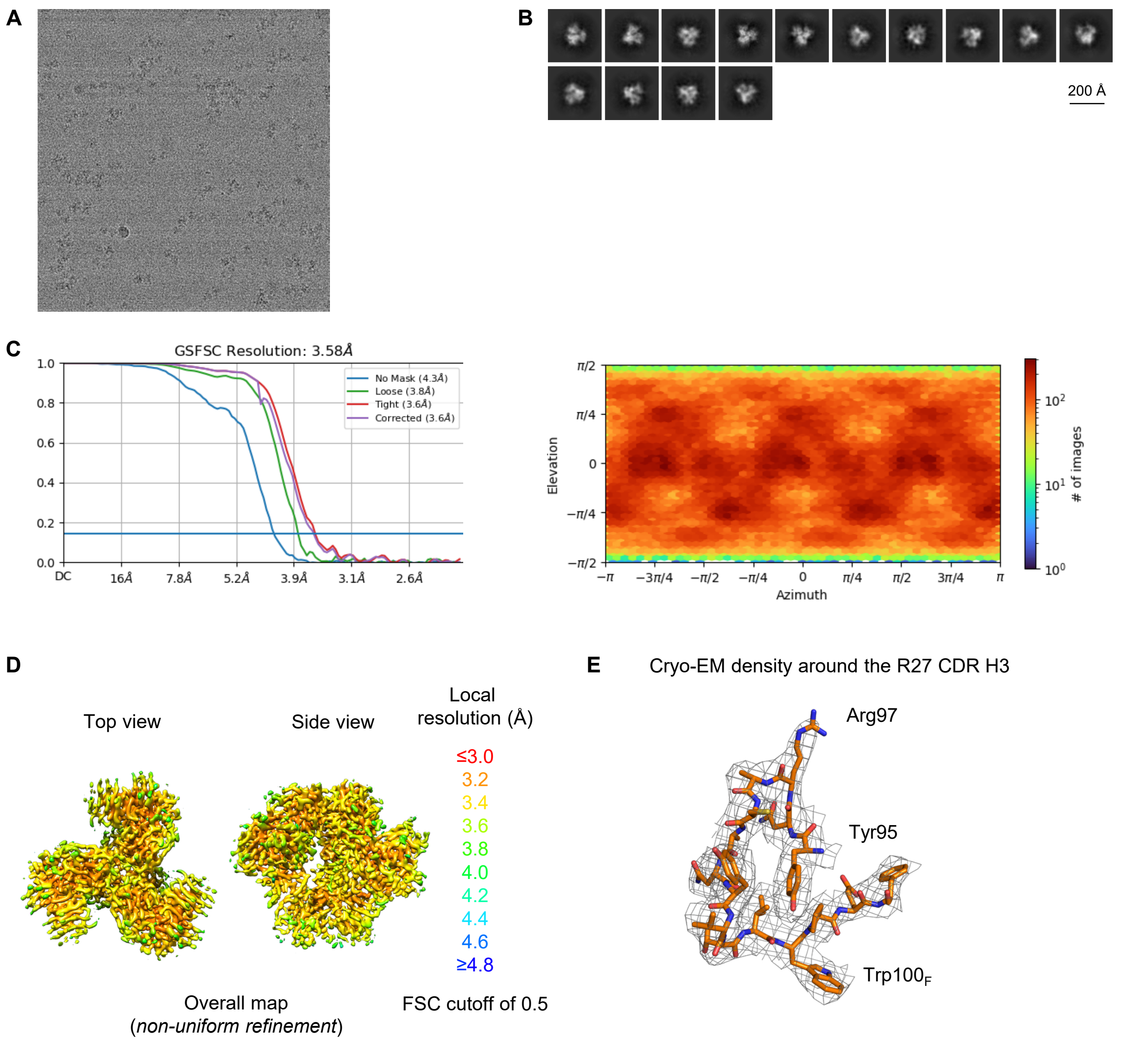

Figure S5. Cryo-EM details of R27 in complex with BG505 DS-SOSIP. (A) Representative micrograph. (B) Representative 2D class averages are shown. (C) The gold-standard Fourier shell correlation resulted in a resolution of 3.58 Å for the overall map using non-uniform refinement with C1 symmetry (left panel); the orientations of all particles used in the final refinement are shown as a heatmap (right panel). (D) The local resolution of the final overall map is shown contoured at 0.809 (9.5 σ). Resolution estimation was generated through cryoSPARC using an FSC cutoff of 0.5. (E) Representative cryo-EM density for the CDR H3 region. The contour level is 6 σ.

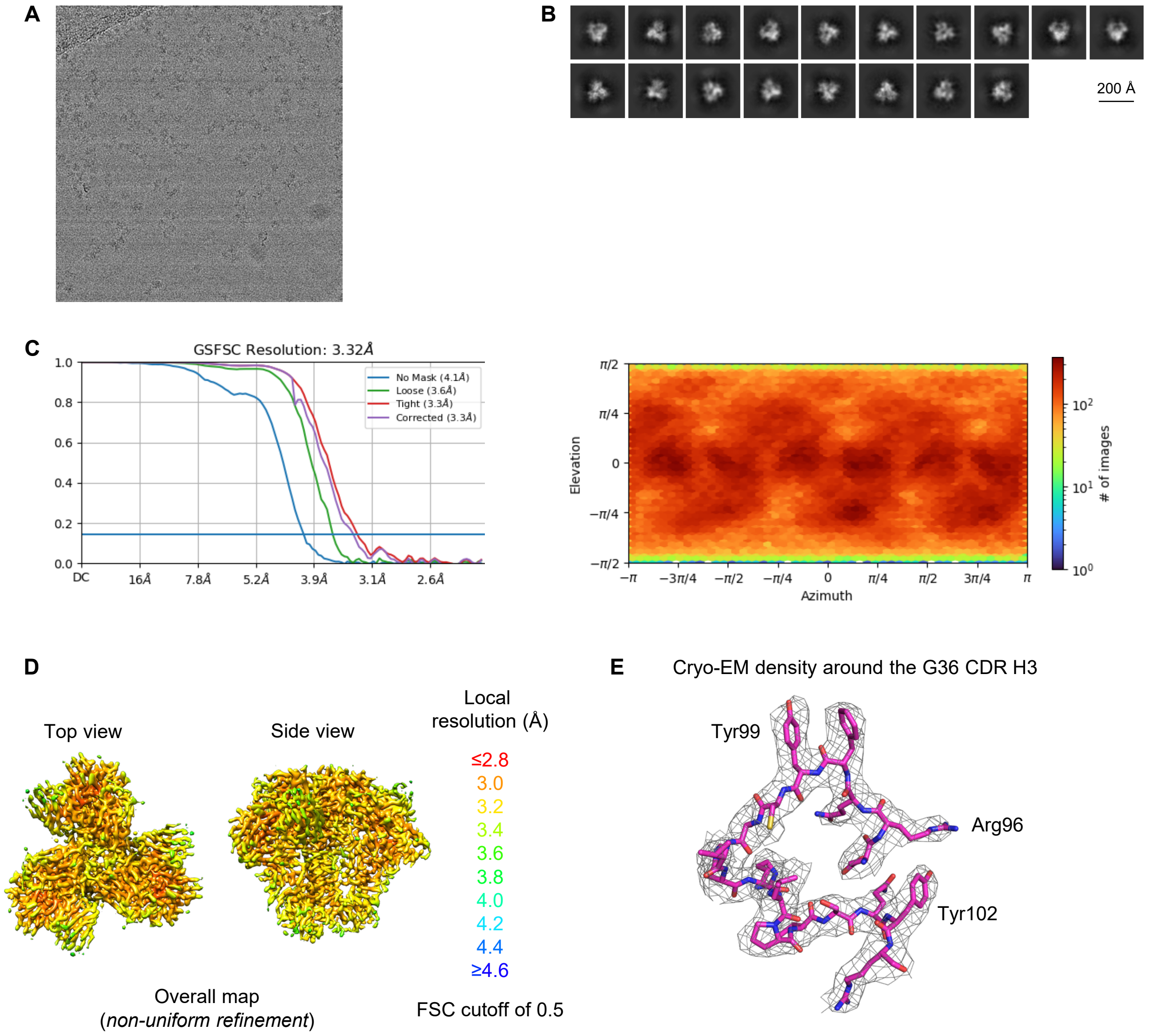

Figure S6. Cryo-EM details of G36 in complex with BG505 DS-SOSIP. (A) Representative micrograph. (B) Representative 2D class averages are shown. (C) The gold-standard Fourier shell correlation resulted in a resolution of 3.32 Å for the overall map using non-uniform refinement with C1 symmetry (left panel); the orientations of all particles used in the final refinement are shown as a heatmap (right panel). (D) The local resolution of the final overall map is shown contoured at 0.956 (9.6 σ). Resolution estimation was generated through cryoSPARC using an FSC cutoff of 0.5. (E) Representative cryo-EM density for the CDR H3 region. The contour level is 5 σ.

A Epitope and paratope surface areas in the R27-BG505 DS SOSIP complex

Epitope (Å^2)		%	Paratope (Å^2)		%
On main protomer	715	97.1	For main protomer	772	97.4
On next protomer	21	2.9	For next protomer	21	2.6
Total	736	100.0		793	100.0

B Hydrogen bonds

	R27	Distance (Å)	HIV-1 Env
1	J:SER 52[OG]	3.78	A:ASP 368[OD2]
2	J:SER 57[OG]	2.64	A:ASP 368[OD2]
3	J:ASN 96[ND2]	3.57	A:GLY 472[O]
4	J:ASN 100a[ND2]	3.25	A:PRO 470[O]
5	J:THR 100d[OG1]	2.76	A:ASP 457[OD2]
6	J:TRP 100f[N]	3.85	A:ASP 457[O]
7	J:ASN 100a[OD1]	3.19	A:SER 364[OG]
8	J:THR 100d[OG1]	3.49	A:ARG 469[NH2]
9	J:THR 100d[OG1]	3.42	A:ARG 469[NE]
10	J:TRP 100f[O]	3.40	A:ASN 280[ND2]
11	J:GLU 101[OE1]	2.77	A:ASN 279[ND2]

C Epitope and paratope residues

<table>
<tr><th>R27</th><th>HSDC</th><th>ASA</th><th>BSA (Å²)</th><th>ΔiG</th><th>gp120</th><th>HSDC</th><th>ASA</th><th>BSA (Å²)</th><th>ΔiG</th></tr>
<tr><td colspan="10">Major protomer</td></tr>
<tr><td>J:ARG45</td><td></td><td>68.82</td><td>25.55||||</td><td>-0.44</td><td>A:ASN279</td><td>H</td><td>73.12</td><td>30.82|||||</td><td>-0.37</td></tr>
<tr><td>J:SER52</td><td></td><td>1.87</td><td>1.51|||||||||</td><td>0.02</td><td>A:ASN280</td><td>H</td><td>58.85</td><td>43.11|||||||</td><td>-0.11</td></tr>
<tr><td>J:LYS53</td><td></td><td>52.74</td><td>0.37|</td><td>0</td><td>A:ALA281</td><td></td><td>80.77</td><td>30.83||||</td><td>0.49</td></tr>
<tr><td>J:ASN54</td><td></td><td>118.67</td><td>80.88|||||||</td><td>-0.39</td><td>A:LYS282</td><td></td><td>64.63</td><td>6.89||</td><td>0.08</td></tr>
<tr><td>J:SER57</td><td>H</td><td>67.71</td><td>36.84|||||</td><td>0.33</td><td>A:ASN283</td><td></td><td>35.7</td><td>5.41||</td><td>-0.09</td></tr>
<tr><td>J:GLY58</td><td></td><td>66.9</td><td>1.80|</td><td>-0.01</td><td>A:SER364</td><td>H</td><td>23.81</td><td>5.87|||</td><td>-0.04</td></tr>
<tr><td>J:ILE59</td><td></td><td>92.75</td><td>4.02|</td><td>0.06</td><td>A:SER365</td><td></td><td>87.54</td><td>52.64||||||</td><td>0.34</td></tr>
<tr><td>J:HIS61</td><td></td><td>71.07</td><td>39.53|||||</td><td>-0.4</td><td>A:GLY366</td><td></td><td>54.97</td><td>31.78|||||</td><td>-0.07</td></tr>
<tr><td>J:ASN96</td><td>H</td><td>91.07</td><td>33.52||||</td><td>-0.4</td><td>A:GLY367</td><td></td><td>69.22</td><td>51.67|||||||</td><td>0.31</td></tr>
<tr><td>J:ARG97</td><td></td><td>95.15</td><td>22.08|||</td><td>0.35</td><td>A:ASP368</td><td>H</td><td>97.49</td><td>75.51|||||||</td><td>-0.29</td></tr>
<tr><td>J:ALA98</td><td></td><td>70.91</td><td>59.52|||||||||</td><td>0.52</td><td>A:VAL371</td><td></td><td>38.66</td><td>34.47|||||||||</td><td>0.55</td></tr>
<tr><td>J:CYS99</td><td></td><td>16.75</td><td>16.75||||||||||</td><td>-0.15</td><td>A:GLN428</td><td></td><td>109.02</td><td>54.79|||||</td><td>0.16</td></tr>
<tr><td>J:ALA100</td><td></td><td>40.8</td><td>31.50|||||||</td><td>0.5</td><td>A:ARG429</td><td></td><td>73.61</td><td>4.39|</td><td>0.04</td></tr>
<tr><td>J:ASN100a</td><td>H</td><td>112.36</td><td>111.29|||||||||</td><td>-0.79</td><td>A:ILE430</td><td></td><td>166.8</td><td>42.17|||</td><td>0.67</td></tr>
<tr><td>J:TYR100b</td><td></td><td>40.3</td><td>27.35||||||</td><td>0.18</td><td>A:THR455</td><td></td><td>30.32</td><td>18.76||||||</td><td>0.26</td></tr>
<tr><td>J:VAL100c</td><td></td><td>68.48</td><td>19.58|||</td><td>0.31</td><td>A:ARG456</td><td></td><td>30.76</td><td>6.02||</td><td>-0.07</td></tr>
<tr><td>J:THR100d</td><td>H</td><td>82.64</td><td>62.42|||||||</td><td>0.14</td><td>A:ASP457</td><td>H</td><td>56.87</td><td>49.68||||||||</td><td>-0.03</td></tr>
<tr><td>J:ILE100e</td><td></td><td>80.9</td><td>57.95|||||||</td><td>0.93</td><td>A:GLY458</td><td></td><td>30.37</td><td>10.49||||</td><td>-0.12</td></tr>
<tr><td>J:TRP100f</td><td>H</td><td>136.63</td><td>68.64|||||</td><td>0.53</td><td>A:GLY459</td><td></td><td>64.03</td><td>33.78|||||</td><td>0.22</td></tr>
<tr><td>J:PRO100g</td><td></td><td>11.21</td><td>4.35||||</td><td>0.07</td><td>A:SER460</td><td></td><td>86.84</td><td>16.68||</td><td>-0.14</td></tr>
<tr><td>J:GLU101</td><td>H</td><td>109.95</td><td>66.40||||||</td><td>-0.21</td><td>A:ARG469</td><td>H</td><td>59.7</td><td>24.74|||||</td><td>-0.19</td></tr>
<tr><td></td><td></td><td></td><td></td><td></td><td>A:PRO470</td><td>H</td><td>6.65</td><td>5.16|||||||</td><td>-0.06</td></tr>
<tr><td></td><td></td><td></td><td></td><td></td><td>A:GLY471</td><td></td><td>10.73</td><td>8.69||||||||</td><td>0.14</td></tr>
<tr><td></td><td></td><td></td><td></td><td></td><td>A:GLY472</td><td>H</td><td>24.36</td><td>15.67||||||</td><td>-0.16</td></tr>
<tr><td></td><td></td><td></td><td></td><td></td><td>A:GLY473</td><td></td><td>53.55</td><td>30.28|||||</td><td>0.42</td></tr>
<tr><td></td><td></td><td></td><td></td><td></td><td>A:ASP474</td><td></td><td>89.06</td><td>24.49|||</td><td>0.2</td></tr>
<tr><td colspan="10">Neighboring protomer</td></tr>
<tr><td>J:PHE 27</td><td></td><td>192.12</td><td>5.18 |</td><td>0.08</td><td>E:THR 63</td><td></td><td>136.69</td><td>5.35 |</td><td>0.09</td></tr>
<tr><td>J:ASP 72</td><td></td><td>130.89</td><td>15.84 ||</td><td>-0.17</td><td>E:LYS 65</td><td></td><td>148.53</td><td>0.34 |</td><td>0.01</td></tr>
<tr><td></td><td></td><td></td><td></td><td></td><td>E:LYS 207</td><td></td><td>139.22</td><td>14.89 ||</td><td>0.24</td></tr>
</table>

Figure S7. Binding surfaces and bonds in the R27-BG505 DS-SOSIP complex. (A) Binding surface areas at the interface of R27 and BG505 DS-SOSIP. R27 contacts two neighboring protomers in the trimeric HIV-1 Env. Listed is data analysis for one R27 (chain J) with its major binding gp120 (chain A) and minor binding gp120 (chain E). (B) Hydrogen bonds between R27 and gp120. (C) Interface residues and their contributions. The data was obtained from the PDBePISA calculation.

A Epitope and paratope surface areas in the G36-BG505 DS SOSIP complex

Epitope ($Å^2$)		%	Paratope ($Å^2$)		%
On main protomer	885	85.2	For main protomer	887	84.9
glycan197	30			32	
On next protomer	172	14.8	For next protomer	163	15.1
Total	1087	100.0		1082	100.0

B Hydrogen bonds and salt bridge

	G36	Distance (Å)	HIV-1 Env
1	J:SER 52[OG]	3.42	A:ASP 368[OD2]
2	J:ASN 54[ND2]	2.87	A:GLU 370[OE2]
3	J:CYS 100[N]	2.61	A:ASP 368[OD2]
4	J:LEU 100b[N]	3.81	A:SER 365[O]
5	J:HIS 100c[ND1]	3.90	A:SER 365[O]
6	J:ARG 100d[NH1]	3.38	A:ARG 456[O]
7	J:ARG 100d[NH2]	2.63	A:ASN 280[O]
		Salt bridge	
1	J:GLU 30[OE2]	3.97	E:LYS 207[NZ]

C Epitope and paratope residues

<table>
<tr><th>G36</th><th>HSDC</th><th>ASA</th><th>BSA ($Å^2$)</th><th>ΔiG</th><th>gp120</th><th>HSDC</th><th>ASA</th><th>BSA ($Å^2$)</th><th>ΔiG</th></tr>
<tr><td colspan="5"></td><td colspan="5">Major protomer</td></tr>
<tr><td>J:GLU30</td><td></td><td>93.21</td><td>28.56||||</td><td>-0.02</td><td>A:ILE194</td><td></td><td>79.35</td><td>55.93||||||||</td><td>0.73</td></tr>
<tr><td>J:SER31</td><td></td><td>55.11</td><td>16.24|||</td><td>0.26</td><td>A:ASN195</td><td></td><td>18.62</td><td>17.59||||||||||</td><td>0.28</td></tr>
<tr><td>J:GLU44</td><td></td><td>140.17</td><td>11.84|</td><td>0.03</td><td>A:THR198</td><td></td><td>110.42</td><td>42.66||||</td><td>0.4</td></tr>
<tr><td>J:SER52</td><td>H</td><td>3.87</td><td>3.87||||||||||</td><td>0.04</td><td>A:SER199</td><td></td><td>24.57</td><td>1.33|</td><td>0.02</td></tr>
<tr><td>J:GLY53</td><td></td><td>10.06</td><td>5.28||||||</td><td>-0.06</td><td>A:ASN279</td><td></td><td>63.43</td><td>0.37|</td><td>-0.01</td></tr>
<tr><td>J:ASN54</td><td>H</td><td>43.18</td><td>38.67|||||||||</td><td>-0.43</td><td>A:ASN280</td><td>H</td><td>77.25</td><td>38.47|||||</td><td>-0.49</td></tr>
<tr><td>J:PHE55</td><td></td><td>180.11</td><td>144.73|||||||||</td><td>2.26</td><td>A:ALA281</td><td></td><td>77.79</td><td>27.16||||</td><td>0.43</td></tr>
<tr><td>J:ASP56</td><td></td><td>84.7</td><td>11.00||</td><td>0.03</td><td>A:LYS282</td><td></td><td>73.18</td><td>9.05||</td><td>0.14</td></tr>
<tr><td>J:GLN57</td><td></td><td>85.74</td><td>25.09|||</td><td>-0.16</td><td>A:SER364</td><td></td><td>29.3</td><td>13.97|||||</td><td>-0.12</td></tr>
<tr><td>J:TYR59</td><td></td><td>96.76</td><td>47.75|||||</td><td>0.4</td><td>A:SER365</td><td>H</td><td>89.68</td><td>40.80|||||</td><td>0.09</td></tr>
<tr><td>J:GLN97</td><td></td><td>106.13</td><td>25.28|||</td><td>-0.15</td><td>A:GLY366</td><td></td><td>74.93</td><td>28.92||||</td><td>0.46</td></tr>
<tr><td>J:PHE98</td><td></td><td>99.6</td><td>76.18||||||||</td><td>1.18</td><td>A:GLY367</td><td></td><td>55.09</td><td>43.12||||||||</td><td>0.15</td></tr>
<tr><td>J:TYR99</td><td></td><td>143.18</td><td>116.79|||||||||</td><td>1.13</td><td>A:ASP368</td><td>H</td><td>110.13</td><td>107.94||||||||||</td><td>-0.5</td></tr>
<tr><td>J:CYS100</td><td>H</td><td>30.28</td><td>28.95||||||||||</td><td>-0.26</td><td>A:LEU369</td><td></td><td>56.4</td><td>0.87|</td><td>-0.01</td></tr>
<tr><td>J:ALA100a</td><td></td><td>48.25</td><td>29.46|||||||</td><td>0.47</td><td>A:GLU370</td><td>H</td><td>69.01</td><td>49.49||||||||</td><td>0.09</td></tr>
<tr><td>J:LEU100b</td><td>H</td><td>64.06</td><td>40.72|||||||</td><td>0.28</td><td>A:VAL371</td><td></td><td>63.47</td><td>49.04||||||||</td><td>0.78</td></tr>
<tr><td>J:HIS100c</td><td>H</td><td>140.41</td><td>95.82|||||||</td><td>0.17</td><td>A:THR372</td><td></td><td>23.94</td><td>2.70||</td><td>-0.03</td></tr>
<tr><td>J:ARG100d</td><td>H</td><td>134.98</td><td>74.30||||||</td><td>-0.12</td><td>A:LYS421</td><td></td><td>28.91</td><td>6.99|||</td><td>-0.13</td></tr>
<tr><td>J:VAL100g</td><td></td><td>104.07</td><td>50.87|||||</td><td>0.79</td><td>A:ILE423</td><td></td><td>12.45</td><td>10.48|||||||||</td><td>0.04</td></tr>
<tr><td>J:GLN101</td><td></td><td>136.68</td><td>15.94||</td><td>-0.1</td><td>A:ILE424</td><td></td><td>13.91</td><td>3.74|||</td><td>0.06</td></tr>
<tr><td></td><td></td><td></td><td></td><td></td><td>A:ASN425</td><td></td><td>74.92</td><td>56.43||||||||</td><td>-0.03</td></tr>
<tr><td></td><td></td><td></td><td></td><td></td><td>A:GLN428</td><td></td><td>117.09</td><td>62.67||||||</td><td>0.2</td></tr>
<tr><td></td><td></td><td></td><td></td><td></td><td>A:ARG429</td><td></td><td>75.02</td><td>9.49||</td><td>0.01</td></tr>
<tr><td></td><td></td><td></td><td></td><td></td><td>A:ILE430</td><td></td><td>165.01</td><td>65.44||||</td><td>1.05</td></tr>
<tr><td></td><td></td><td></td><td></td><td></td><td>A:GLN432</td><td></td><td>82.5</td><td>0.86|</td><td>-0.01</td></tr>
<tr><td></td><td></td><td></td><td></td><td></td><td>A:ALA433</td><td></td><td>22.34</td><td>8.87||||</td><td>0.14</td></tr>
<tr><td></td><td></td><td></td><td></td><td></td><td>A:THR455</td><td></td><td>41.52</td><td>33.90|||||||||</td><td>0.52</td></tr>
<tr><td></td><td></td><td></td><td></td><td></td><td>A:ARG456</td><td>H</td><td>34.99</td><td>6.85||</td><td>-0.08</td></tr>
<tr><td></td><td></td><td></td><td></td><td></td><td>A:ASP457</td><td></td><td>65.35</td><td>12.11||</td><td>-0.08</td></tr>
<tr><td></td><td></td><td></td><td></td><td></td><td>A:GLY459</td><td></td><td>70.24</td><td>12.15||</td><td>-0.09</td></tr>
<tr><td></td><td></td><td></td><td></td><td></td><td>A:ARG469</td><td></td><td>78.68</td><td>30.48||||</td><td>0.43</td></tr>
<tr><td></td><td></td><td></td><td></td><td></td><td>A:PRO470</td><td></td><td>5.19</td><td>4.78||||||||||</td><td>-0.05</td></tr>
<tr><td></td><td></td><td></td><td></td><td></td><td>A:GLY471</td><td></td><td>17.08</td><td>3.52|||</td><td>0.06</td></tr>
<tr><td></td><td></td><td></td><td></td><td></td><td>A:GLY473</td><td></td><td>28.64</td><td>12.79|||||</td><td>0.19</td></tr>
<tr><td></td><td></td><td></td><td></td><td></td><td>A:ASP474</td><td></td><td>73.51</td><td>13.89||</td><td>0.09</td></tr>
<tr><td colspan="5">Residues interacting with glycan197*</td><td colspan="5">Glycan 197</td></tr>
<tr><td>H:ASP 56</td><td></td><td>82.80</td><td>18.39 |||</td><td>-0.31</td><td>Glycan 197</td><td></td><td>357.94</td><td>30.26 |</td><td>-1.00</td></tr>
<tr><td>H:ASN 74</td><td></td><td>91.36</td><td>13.25 ||</td><td>-0.15</td><td></td><td></td><td></td><td></td><td></td></tr>
<tr><td colspan="5"></td><td colspan="5">Neighboring protomer</td></tr>
<tr><td>J:GLN1</td><td></td><td>196.99</td><td>26.51||</td><td>-0.3</td><td>E:GLU62</td><td></td><td>134.99</td><td>19.62||</td><td>0.03</td></tr>
<tr><td>J:GLY26</td><td></td><td>58.08</td><td>4.22|</td><td>0.06</td><td>E:THR63</td><td></td><td>129.31</td><td>8.53|</td><td>0.14</td></tr>
<tr><td>J:ASP28</td><td></td><td>74.15</td><td>7.70||</td><td>0.12</td><td>E:PRO206</td><td></td><td>51.37</td><td>2.01|</td><td>0.03</td></tr>
<tr><td>J:GLU30</td><td>S</td><td>93.21</td><td>9.45||</td><td>-0.11</td><td>E:LYS207</td><td>S</td><td>148.73</td><td>77.37||||||</td><td>-0.47</td></tr>
<tr><td>J:ASN74</td><td></td><td>89.4</td><td>7.75|</td><td>-0.08</td><td></td><td></td><td></td><td></td><td></td></tr>
<tr><td>J:GLU75</td><td></td><td>163.49</td><td>95.10||||||</td><td>-0.17</td><td></td><td></td><td></td><td></td><td></td></tr>
<tr><td>J:ASN77</td><td></td><td>43.4</td><td>11.78|||</td><td>-0.03</td><td></td><td></td><td></td><td></td><td></td></tr>
</table>

Figure S8. Binding surfaces and bonds in the G36-BG505 DS-SOSIP complex. (A) Binding surface areas at the interface of nanobody G36 and BG505 DS-SOSIP. G36 contacts two neighboring protomers in the trimeric HIV-1 Env. Listed is data analysis for one G36 (chain J) with its major binding gp120 (chain A) and minor binding gp120 (chain E). (B) Hydrogen bonds between G36 and gp120. (C) Interface residues and their contributions. Listed is data analysis for one G36 (chain J) with its major binding gp120 (chain A) and minor binding gp120 (chain E). * Glycan 197 interaction data is from chain H and gp120 (chain C) with showed better cryo-EM density for this region. The data was obtained from the PDBePISA calculation.

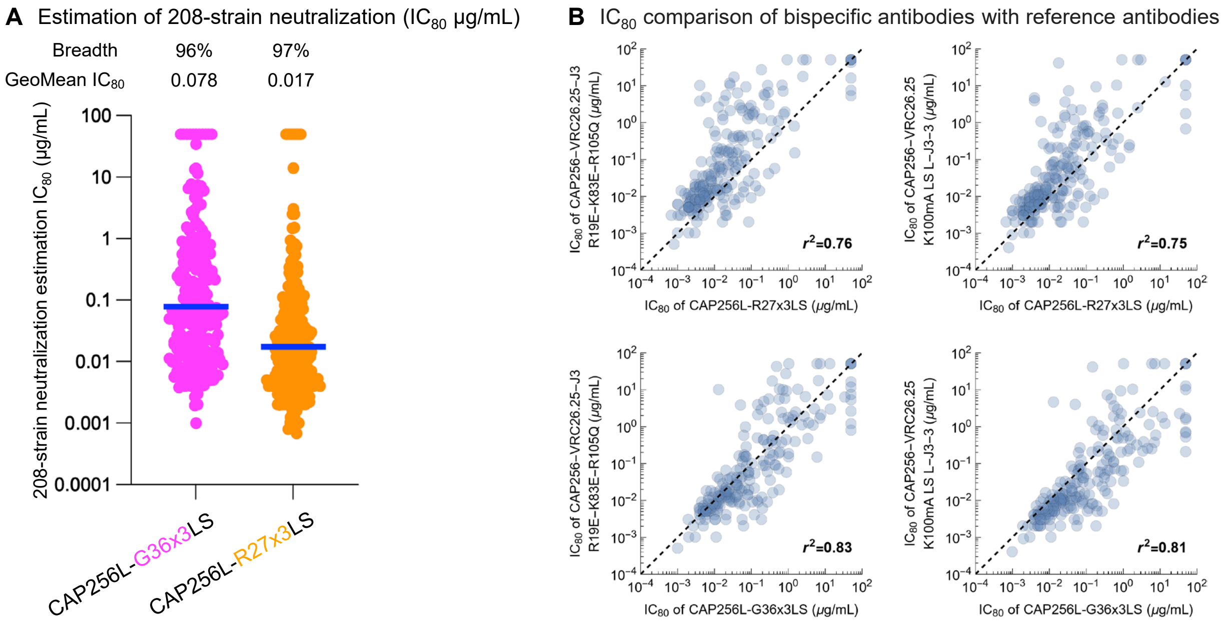

Figure S9. Estimation of 208-strain neutralization of CAP256L-G36x3LS and CAP256L-R27x3LS. (A) Estimated IC_{80} of CAP256L-G36x3LS and CAP256L-R27x3LS. (B) IC_{80} comparison of the two bispecific antibodies with two reference antibodies.

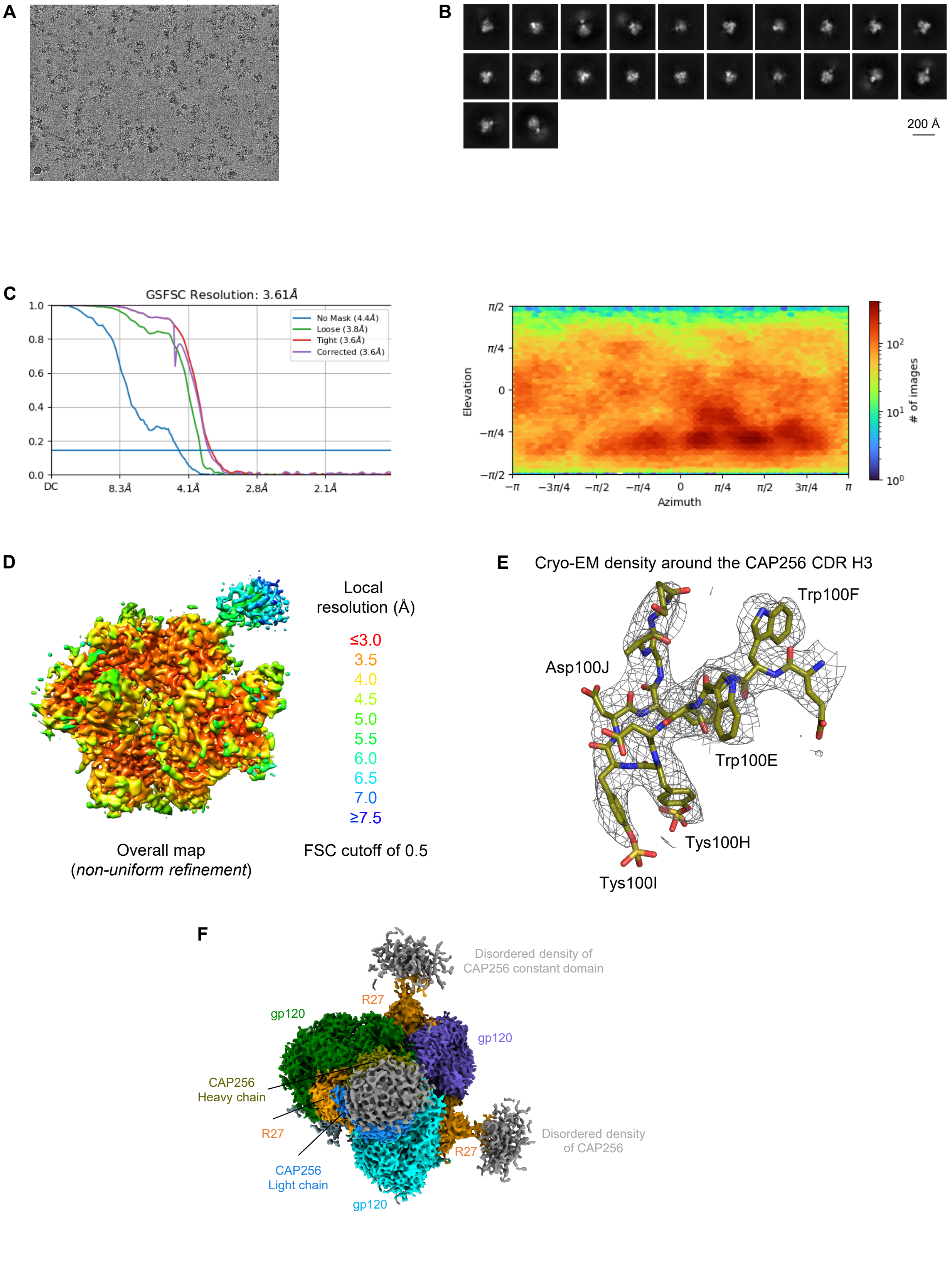

Figure S10. Cryo-EM details of CAP256L-R27LS Fab in complex with BG505 DS-SOSIP. (A) Representative micrograph. (B) Representative 2D class averages are shown. (C) The gold-standard Fourier shell correlation resulted in a resolution of 3.61 Å for the overall map using non-uniform refinement with C1 symmetry (left panel); the orientations of all particles used in the final refinement are shown as a heatmap (right panel). (D) The local resolution of the final overall map is shown contoured at 0.33 (8.1 σ). Resolution estimation was generated through cryoSPARC using an FSC cutoff of 0.5. (E) Representative cryo-EM density of CAP256 CDR H3 region. The contour level is 4.5 σ. (F) Cryo-EM density of the CAP256L-R27-BG505 DS SOSIP complex at low contour (2.5 σ) showing extra disordered density connecting to two R27s, indicating these two R27s were from separate CAP256L-R27 molecules. The density was colored the same as in Figure 7 with un-modeled density colored gray.

Table S1. Cryo-EM Data Collection, Refinement and Validation Statistics for R27, G36 and CAP256L-R27 in complex with HIV-1 BG505 DS-SOSIP.

	R27 in complex with HIV-1 BG505 DS-SOSIP (EMD-41415) (PDB 8TNG)	G36 in complex with HIV-1 BG505 DS-SOSIP (EMD-41416) (PDB 8TNH)	CAP256L-R27 in complex with HIV-1 BG505 DS-SOSIP (EMD-41417) (PDB 8TNI)
Data collection and processing			
Magnification	105,000	105,000	105,000
Voltage (kV)	300	300	300
Electron exposure ($e^{-}/Å^{2}$)	40.0	40.0	58
Defocus range (μm)	-1.0 to -2.5	-1.0 to -2.5	-1.0 to -2.5
Pixel size (Å)	1.110	1.110	0.830
Symmetry imposed	C3	C3	C1
Final particle images (no.)	315969	414002	236154
Map resolution (Å)	3.58	3.32	3.61
FSC threshold	0.143	0.143	0.143
Refinement			
Initial model used (PDB code)	7LPN	7LPN	8FIS
Model resolution (Å)	3.61	3.61	3.18
FSC threshold	0.5	0.5	0.5
Map sharpening B factor ($Å^{2}$)	-217.9	-200.7	-127.9
Model composition			
Non-hydrogen atoms	17499	17690	19408
Protein residues	2101	2113	2358
Ligands	75	75	72
B factors ($Å^{2}$)(mean)			
Protein	82.9	86.7	72.1
Ligand	103.7	94.7	83.0
R.m.s. deviations			
Bond lengths (Å)	0.003	0.004	0.004
Bond angles (°)	0.719	0.657	0.839
Validation			
MolProbity score	2.04	1.87	2.24
Clash score	8.9	6.0	14.0
Poor rotamers (%)	0.60	0.43	0.39
Ramachandran plot			
Favored (%)	89.5	90.4	88.6
Allowed	10.0	9.6	11.0
Disallowed	0.5	0	0.4
